# A simple and efficient CRISPR/Cas9 platform for induction of single and multiple, heritable mutations in barley (*Hordeum vulgare* L.)

**DOI:** 10.1186/s13007-018-0382-8

**Published:** 2018-12-18

**Authors:** Sebastian Gasparis, Maciej Kała, Mateusz Przyborowski, Leszek A. Łyżnik, Wacław Orczyk, Anna Nadolska-Orczyk

**Affiliations:** 1 Department of Functional Genomics, Plant Breeding and Acclimatization Institute – National Research Institute, 05-870 Radzików, Błonie, Poland; 2 Department of Plant Genetics, Breeding and Biotechnology, Warsaw University of Life Sciences (SGGW), 02-776 Warsaw, Poland; 3 Department of Genetic Engineering, Plant Breeding and Acclimatization Institute – National Research Institute, 05-870 Radzików, Błonie, Poland

**Keywords:** CRISPR/Cas9, Genome editing, Barley, PTG, *CKX* genes

## Abstract

**Background:**

Genome editing of monocot plants can be accomplished by using the components of the CRISPR/Cas9 (clustered regularly interspaced short palindromic repeat/CRISPR associated Cas9) technology specifically optimized for these types of plants. Here, we present the development of RNA-guided Cas9 system for simplex and multiplex genome editing in barley.

**Results:**

We developed a set of customizable RNA-guided Cas9 binary vectors and sgRNA modules for simplex and multiplex editing in barley. To facilitate the design of RNA-guided Cas9 constructs, the pBract derived binary vectors were adapted to Gateway cloning and only one restriction enzyme was required for construction of the sgRNA. We designed a synthetic, codon optimized Cas9 gene containing the N terminal SV40 nuclear localization signal and the UBQ10 *Arabidopsis* 1st intron. Two different sgRNAs were constructed for simplex editing and one polycistronic tRNA-gRNA construct (PTG) for multiplex editing using an endogenous tRNA processing system. The RNA-guided Cas9 constructs were validated in transgenic barley plants produced by *Agrobacterium*-mediated transformation. The highest mutation rate was observed in simplex editing of the cytokinin oxidase/dehydrogenase *HvCKX1* gene, where mutations at the *hvckx1* locus were detected in 88% of the screened T_0_ plants. We also proved the efficacy of the PTG construct in the multiplex editing of two *CKX* genes by obtaining 9 plants (21% of all edited plants) with mutations induced in both *HvCKX1* and *HvCKX3*. Analysis of the T_1_ lines revealed that mutations in the *HvCKX1* gene were transmitted to the next generation of plants. Among 220 screened T_1_ plants we identified 85 heterozygous and 28 homozygous mutants, most of them bearing frameshift mutations in the *HvCKX1* gene. We also observed independent segregation of mutations and the Cas9-sgRNA T-DNA insert in several T_1_ plants. Moreover, the knockout mutations of the *Nud* gene generated phenotype mutants with naked grains, and the phenotypic changes were identifiable in T_0_ plants.

**Conclusions:**

We demonstrated the effectiveness of an optimized RNA-guided Cas9 system that can be used for generating homozygous knockout mutants in the progeny of transgenic barely plants. This is also the first report of successful multiplex editing in barley using a tRNA processing system.

**Electronic supplementary material:**

The online version of this article (10.1186/s13007-018-0382-8) contains supplementary material, which is available to authorized users.

## Introduction

The progress in DNA sequencing technology with increasing speed, scalability at relatively low cost combined with advanced bioinformatics led to accumulation of immense amount of data on genomic sequences, annotated genes and transcriptome profiles of large number of diverse species. The knowledge of plant genomes opens new possibilities for both genetic studies and crop improvement by genetic engineering techniques. However, this knowledge cannot be fully utilized without appropriate research tools for fast and accurate structural/functional analysis and annotation of the sequenced genes. The utilization of various reverse genetics concepts had been limited until the emergence of a breakthrough genome editing technology based on the application of programmable sequence specific endonucleases. In practice, an endonuclease can be modified to acquire specific DNA binding domains and then transferred to the nucleus where it generates double strand DNA breaks (DSB) in a site-specific manner (reviewed by Puchta and Fauser [1]). Until 2013, the most popular genome editing procedures utilized zinc finger nucleases (ZFN) [2] and transcription activator-like effector nucleases (TALENs) [3, 4]. ZFNs and TALENs have not been widely adopted due to the complexities of their design and synthesis. The emergence of the CRISPR/Cas9 (clustered regularly interspaced short palindromic repeat/Cas9-associated) system has revolutionized genome editing technologies. Simplicity, efficiency, and versatility of this system makes it a popular genetic tool. It was adapted from the prokaryotic adaptive immunity systems, which act against invading foreign DNA [5]. Unlike *Fok*I-based site specific nucleases, Cas9 endonucleases are monomeric proteins with two nuclease domains that cut DNA 3 bp upstream of the PAM motif (Protospacer Adjacent Motif). The specificity is conferred by a 20 nt fragment of gRNA (guide RNA), which binds to the complementary target sequence (protospacer). The presence of a double strand break generated by Cas9 induces intracellular mechanisms of DNA repair, which can be divided into two major groups: homology-directed repair (HDR) and non-homologous end joining (NHEJ). In the HDR mechanism, a broken DNA molecule is repaired via homologous recombination; therefore, the template DNA is required to restore the original sequence or to introduce a specific mutation. In the NHEJ mechanism the free ends of DNA are joined via non-homologous (illegitimate) recombination. As a result of errors in the DSB repair process, small insertions, deletions, or rearrangements may occur at the joining site. If the DSB is generated inside the open reading frame, the error-prone DSB repair may lead to knockout mutations. In somatic cells the NHEJ is a predominant repair mechanism, as opposed to meiotic cells, where DSBs are repaired by homologous recombination [6]. The sequences of Cas9 and sgRNA (single guide RNA) are the only elements of the CRISPR/Cas9 system, which need to be expressed in host cells They can be introduced on a single T-DNA construct. Moreover, the CRISPR/Cas9 system can be used for multiplex editing of different target sequences, as multiple sgRNAs can be transcribed from a single polycistronic gene [7].

The CRISPR/Cas9 system was initially tested in model plants such as *Arabidopsis*, tobacco, and rice [8–14]. Thereafter, the studies were extended on the application of CRISPR/Cas9 to crop species. Since then, various RNA-guided Cas9 platforms have been developed for more efficient editing and to facilitate the technical aspects of the construction of custom RNA-guided Cas9 vectors (reviewed in [15–17]). In cereals, the studies involving the RNA-guided Cas9 system were focused mainly on three of the most important species: rice [7, 11, 13, 18–24], maize [25–28] and wheat [29–32].

With its sequenced diploid genome, barley may be considered as a diploid model species for closely related hexaploid cereals. There are two reports of RNA-guided Cas9 based genome editing in this crop plant. Lawrenson et al. [33] investigated the target specificity of the RNA-guided Cas9 system in barley by targeting two copies of the *HvPM19* gene and observed induced mutations in 23% and 10% of the T_0_ lines, respectively. The mutations were stably transmitted to T_2_ plants independently of the T-DNA construct. Moreover, the authors reported off-target mutations in non-target copies of *HvPM19*. Kapusi et al. [34] used five different sgRNA constructs for targeting the ENGase gene in barley by both biolistic and *Agrobacterium*-mediated transformation. Mutations in the ENGase gene were detected in 25 out of 32 primary transformants in seven lines produced by particle bombardment and 18 lines derived from *Agrobacterium*-transformation.

For our studies, we choose *HvCKX1* and *HvCKX3*, two genes belonging to a small family of genes encoding cytokinin oxidase/dehydrogenase enzymes that regulate endogenous levels of cytokinin hormones [35, 36]. The *CKX* genes show diversified developmental and tissue specific expression patterns [37, 38], suggesting their specialized functions in diverse plant tissues. The *CKX1* and *CKX3* knock-out lines may serve as interesting objects for further studies. The function of the ethylene response transcription factor gene *Nud* is known (it controls the formation of hulled grains [39]), and therefore, we chose it to validate the efficacy of our RNA-guided Cas9 constructs in generating phenotypic changes in barley mutant plants.

Here, we present analternative version of the RNA-guided Cas9 system designed for genome editing in barley. A relatively high rate of induced mutations was achieved by using a synthetic, codon optimized, and intron-enhanced Cas9 gene in combination with an efficient *Agrobacterium*-transformation method. The design of the binary vectors was also optimized to minimize the cloning steps. Only one restriction enzyme *Bsa*I was required for construction of the sgRNA cassette, which was subsequently integrated into the Cas9 binary vector by Gateway cloning. Using our RNA-guided Cas9 constructs we were able to produce T_0_ plants with visible phenotypic changes and homozygous, transgene-free mutants could be selected in the T_1_ generation. This is also the first report of multiplex editing in barley using an endogenous tRNA processing system.

## Methods

### Design of the Cas9 gene for expression in barely cells

The sequence of the Cas9 gene from *Streptococcus pyogenes* (Gene Bank accession AMH03982) was optimized for expression in monocot plants using the GeneOptimizer program (Thermo Fisher Scientific). The GC content was increased from 35.1 to 56.5% and the amino acid codon usage was adjusted to that observed in maize (https://www.kazusa.or.jp/codon). In order to facilitate translocation of the Cas9 endonuclease protein from cytoplasm to nucleus, a version of the SV40 (simian virus 40) large T antigen NLS (nuclear localization signal) (NLS-SV40, MAPKKKRKVG) sequence was added to the N-terminus of the Cas9 protein. The Arabidopsis’ 310 nt UBQ10 1st intron was placed at + 196 within an AGGT canonical splice site sequence of the Cas9 coding sequence. The UBQ10 1st intron is among the best studied enhancing introns in plants [40]. The enhancing effect of this intron in conjunction with the maize ubi promoter was tested in barely plants, while integrated at the + 165 position in the luciferase gene used for testing the enhancement effect [41]. An additional restriction site—*Nco*I— was also incorporated into the 5′ flanking sequence together with the consensus Kozak’s sequence (CCGCCATGG) around the ATG start codon. Three internal *Sac*I and one *Hin*dIII restriction sites were mutagenized, while preserving the amino acid sequence of the Cas9 protein.

The complete nucleotide sequence of Cas9 is shown in the Additional file 1: Methods S1. Construction of the RNA-guided Cas9 vector.

For cloning Cas9 into the pBract211 vector, the *Sph*I restriction site was removed and two unique restriction sites for the *Xma*I and *Spe*I enzymes were added to both sides of the Cas9 coding sequence. The resulting 4464 bp Cas9 sequence was synthesized and cloned into the *Xma*I/*Spe*I restriction site of the pBract211 vector [42]. The pBract211 vector containing the Cas9 gene was then converted to a Gateway compatible destination vector (pBract211-Cas-GW) using the Gateway conversion kit (Invitrogen). The vector was cut at the *Sph*I site and the Gateway cassette was cloned directly after the *nos* terminator. This site is used for Gateway cloning of a sgRNA cassette with a chosen target sequence. The detailed cloning protocol of the RNA-guided Cas9 vector is described in Additional file 1: Methods S1.

### Design of the sgRNA

**Fig. 1:**
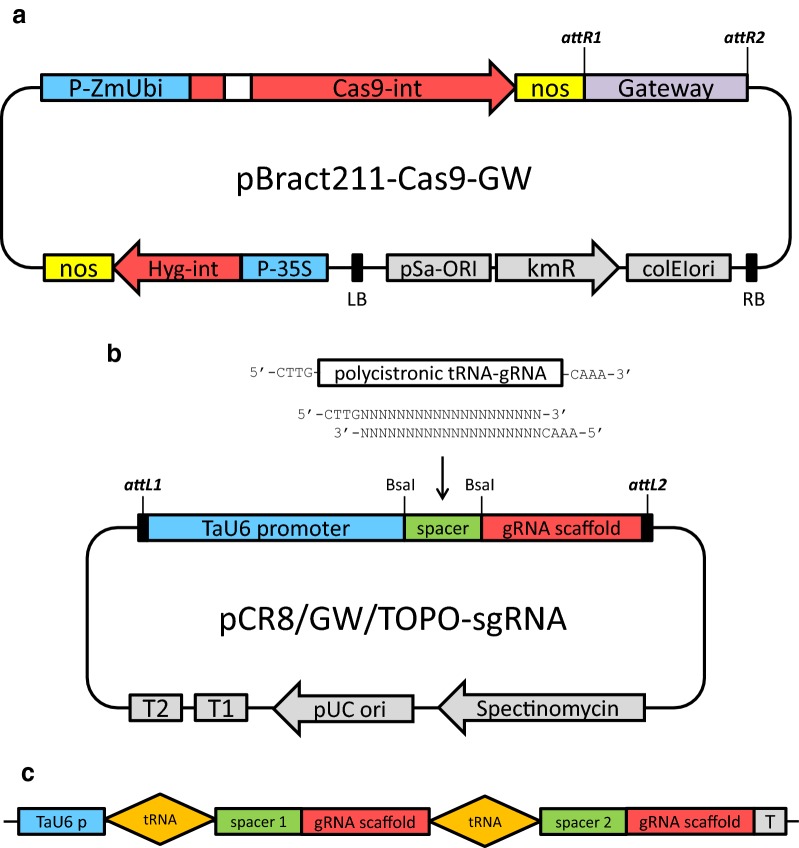
Schematic description of RNA-guided Cas9 constructs designed for genome editing. **a** Structure of the binary vector plasmid based on pBract211 used to deliver Cas9:sgRNA components into barley plants. The Gateway cloning site is replaced by the sgRNA cassette; P-ZmUbi, maize ubiquitin promoter; Cas9-int, synthetic gene of nuclease Cas9 with an intron and nuclear localization signal; *nos*, nopaline synthase terminator; P-35S, CaMV35S promoter; *Hyg*-int, hygromycin resistance gene; *kmR*, kanamycin resistance gene. **b** Structure of the pCR8/GW/TOPO-sgRNA vector used for assembling the sgRNA or PTG constructs. Either oligo duplex for simplex editing or polycistronic tRNA-gRNA (PTG) for multiplex editing can be cloned between the U6 promoter and gRNA scaffold using *Bsa*I-generated overhangs. **c** Structure of the polycistronic tRNA-gRNA unit with two spacers used for multiplex editing

The sequences of the wheat U6 promoter (GenBank accession X63066.1) and gRNA scaffold [13] were synthesized as a single sequence containing a short insert between them with two *Bsa*I restriction sites in the opposite orientation (see Additional file 1: Methods S1 for the complete sequence). To facilitate the detection of induced mutations each target sequence contained the restriction site overlapping the cutting site generated by Cas9 3 bp upstream of the PAM motif. The target sequence in the form of two annealed oligos was cloned between the U6 promoter and gRNA scaffold at the *Bsa*I cleavage site. The target sequence of the annealed oligos was forward 5′-CTTG(N)_20_-3′ and reverse 5′-AAAC(N)_20_-3′ (Fig. 1, Additional file 1: Methods S1). The detailed protocol of oligo annealing and sgRNA ligation, and the target sequences used in this study are described in the Additional file 1: Methods S1 and Table S2. The sgRNA cassette was then cloned into the Gateway entry vector. We used the pCR8/GW/TOPO vector, however any Gateway compatible entry vector with attL1/attL2 sites could be used.

### Design of the PTG cassette

A polycistronic tRNA-gRNA cassette was designed for a simultaneous targeting of the *HvCKX1* and *HvCKX3* genes using a single U6-gRNA construct. The sgRNA elements were flanked by glycine-tRNA sequences from barley (ASM32608v1:7:24838229:24838299:1) and cloned between the TaU6 promoter and gRNA scaffold after cutting it with the *Bsa*I restriction enzyme. The sequences of TaU6 promoter and gRNA scaffold were the same as for sgRNA. The sequence of the PTG construct is shown in Table S2 and the detailed assembly protocol is described in Additional file 1: Methods S1.

### *Agrobacterium*-mediated transformation

Immature embryos of barley cv Golden Promise were transformed using the protocol of Harwood et al. [43] with modifications. The donor plants were grown in a controlled environmental chamber at 18 °C day and 12 °C night temperatures, and a 16 h photoperiod with 350 μmol/m^2^/s light intensity provided by fluorescent lamps. The same conditions were applied to regenerated transgenic T_0_ plants. The immature embryos were isolated from surface-sterilized caryopses, and after excising the embryonic axes were placed on callus induction medium (CI) (see Additional file1: Table S3 for medium composition) scutellum side up. On the same day the embryos were inoculated with *Agrobacterium tumefaciens* culture with the addition of 200 µM of acetosyringone. The *Agrobacterium* culture was grown overnight on MGL medium without antibiotics at 28 °C. Directly after inoculation the embryos were transferred to fresh CI medium, scutellum side down, and co-cultivated for 3 days in the dark. After co-cultivation the embryos were transferred to fresh CI plates containing hygromycin as a selective agent and Timentin to eliminate *Agrobacterium* from the culture. The embryos and emerging calli were cultured at 22–23 °C in the dark for 6 weeks, and passed to fresh selection CI medium every 2 weeks. Embryo-derived calli were transferred to the transition medium (TR) and incubated at 22–23 °C for 2–3 weeks under low light conditions that were achieved by covering the plates with two sheets of filter paper. Small regenerating shoots, which emerged during this time, were transferred to regeneration medium (Reg) and cultured under full light conditions until they formed 2–3 cm plantlets with small roots. The plantlets were next transferred from plates to glass jars with half-strength MS medium without growth regulators but still containing hygromycin and Timentin. Plants, which formed a strong root system, were planted into soil. For the first 2 weeks the plants in the pots were covered with transparent glass jars for acclimation to new conditions. The putative transgenics were PCR screened for the presence of a T-DNA fragment using primers specific to the *hpt* gene (see Additional file 1: Table S1).

### Detection of mutations induced by RNA-guided Cas9

For each PCR-positive transgenic plant leaf tissue samples were collected for extraction of genomic DNA. Tissue fragments were collected from possibly every leaf to minimize the risk of false negatives in case of chimeric plants. Genomic DNA was extracted using a standard CTAB method [44]. The target genes were amplified with specific primers flanking the designed target sequence (see Additional file 1: Table S1 for primer sequences) using Q5 Hot-start polymerase (NEB). After amplification, 10 µl of the PCR mixture was taken for restriction digestion with the *Bsm*AI (*HvCKX1* and *Nud*) or *Ban*II (*HvCKX3*) enzymes. The digested amplicons were separated on 1.5% agarose gels and imaged on a Kodak Gel Logic 200 Imaging System. PCR products from samples with mutations detected on the gel were cloned into the pGEM-T Easy vector (Promega) for sequencing. At least 10 clones from each sample were sequenced.

### Statistical analysis

The confidence level for segregation ratios of T-DNA in T_1_ lines was calculated in Excel spreadsheet using Chi squared test.

## Results

Strategy for simplex and multiplex genome editing of barley based on an optimized Cas9 and gRNA module.

An optimized synthetic Cas9 gene based on the native sequence from *Streptococcus pyogenes* was used for mutagenesis experiments in barely cells. The optimization process included the adjustment of the coding sequence GC content and amino acid codon usage according to that observed in monocot plants. In addition to other minor modifications, the 310 bp UBQ10-i1 intron from *Arabidopsis thaliana* was placed within the 5′ coding region of Cas9. The recombined Cas9-encoding gene was cloned to the pBract211-derived binary vector for plant genetic transformation and the resulting construct was designated as pBract211-Cas9 (Fig. 1a).

To introduce sgRNA, the second component of the RNA-guided Cas9 system, the pBract211-Cas9 vector was converted into the Gateway destination vector.

The Gateway cassette containing the *att*R1 and *att*R2 recombination sites was placed directly after the *nos* terminator within the T-DNA fragment (Fig. 1a, Additional file 1: Methods S1).

Construction of this intermediate vector facilitated the next cloning step, in which the complete sgRNA cassette consisting of the U6 or U3 promoter, spacer, gRNA scaffold, and U6 or U3 terminator was introduced into the vector by Gateway LR reaction.

We decided to use this method for two reasons: (1) to avoid restriction cloning of both the sgRNA and spacers directly into the pBract211 vector with incompatible restriction enzymes and (2) to facilitate the production of multiple sets of pBract211-Cas9 vectors with different variants of sgRNA cassettes (e.g. for simplex or multiplex editing, or with different RNA promoters). This ensures more versatility of our system, as one can use the existing gRNA constructs, which are shared in repositories or exchanged between laboratories.

The sgRNA module for simplex editing was designed similar to the strategy described by Shan et al. [13]. The DNA fragment consisting of the U6 RNA promoter from wheat and the sgRNA sequence with transcription termination signal was synthesized and cloned into the pCR8/GW/TOPO intermediate vector. The final pCR8/GW/TOPO Gateway entry vector was obtained by inserting a 20 bp spacer complementary to the target sequence between the U6 promoter and gRNA scaffold. The spacer was inserted as an annealed oligo using type-II restriction endonucleases which produces minimal 4 nt overhangs (Fig. 1b, Fig. S2).

We decided to use *Bsa*I instead of *Bbs*I, as this enzyme is compatible with Golden Gate cloning vectors. The RNA transcripts driven by the U6 promoter start with G, therefore the optimal target sequence should conform to the pattern G(N)_19_NGG, however, it is not obligatory as the (N)_20_NGG spacers also work.

For multiplex editing we decided to use the tRNA processing system-based strategy proposed by Xie et al. [7]. This mechanism can be used to simultaneously produce multiple gRNAs from a single polycistronic tRNA-gRNA gene (PTG). Based on this strategy we assembled a PTG gene with two different gRNAs fused with two glycine-tRNA sequences from barley (ASM32608v1:7:24838229:24838299:1) (Fig. 1c, Additional file 1: Table S2). Both sgRNA cassettes and the PTG gene were cloned into the pBract211-Cas9 binary vector. In summary, we prepared a set of three RNA-guided Cas9 transformation vectors, two of them for simplex editing and one with the PTG cassette for multiplex editing. As targets we chose three barley genes, *HvCKX1*, *HvCKX3,* and *Nud*. Mutated *Nud* alleles (*nud*) produce naked (hulles) grains with non-adhering hulls, thus the *Nud* gene was targeted to generate mutants with naked grains.

Validation of the RNA-guided Cas9 vectors for simplex and multiplex editing in transgenic barley plants.

**Fig. 2:**
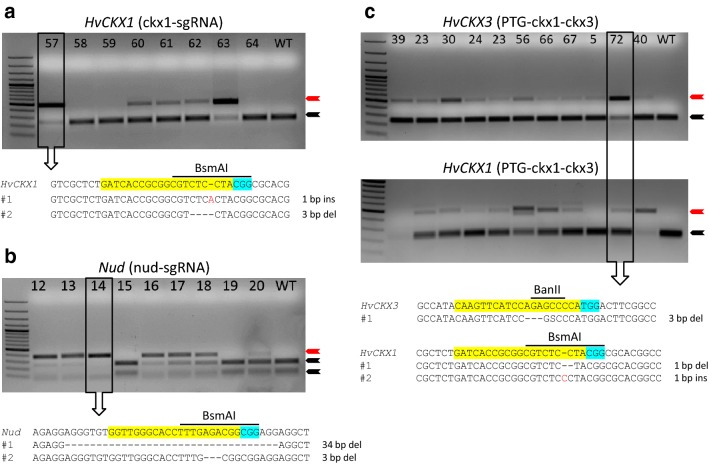
PCR/RE screening of T_0_ plants transformed with Cas9:sgRNA constructs for simplex editing of *HvCKX1* (**a**), *Nud* (**b**), and the PTG construct for multiplex editing of the *HvCKX1* and *HvCKX3* genes (**c**). PCR products of the appropriate target gene were treated by restriction enzymes that overlap the potential mutation site (indicated above the aligned sequences). Red arrowheads indicate uncut bands of amplicons with mutations; black arrowheads indicate bands of wild-type sequences cut by an enzyme. PCR products from selected samples were cloned and sequenced to identify the pattern of mutations; target sequences for sgRNA:Cas9 are marked in yellow, PAM motifs are marked in light blue; deletions are indicated by dashes and insertions by red letters

To test the efficiency of our RNA-guided Cas9 constructs in generating targeted mutations at a single genomic site (locus), we produced transgenic barley plants after *Agrobacterium*-transformation with two vectors, pBract211-Cas9-ckx1 and pBract211-Cas9-nud with gRNAs targeting the *HvCKX1* and *Nud* genes, respectively. The target sequences were chosen near the 5′ end of the ORF to increase the chance of reading frame disruption caused by induced mutations, and we selected those where the cutting site of Cas9 was overlapped by a restriction site. After transformation, all independent transgenic T_0_ plants were screened by PCR amplification and restriction analysis (PCR/RE) of the amplified fragments for mutations at the target sequences. Since most indels generated by the NHEJ repair system should disrupt the restriction site overlapping the double-strand break site, the mutation is confirmed by the presence of an uncut band on an agarose gel, while the wild-type copies of the gene are cut by the enzyme (Fig. 2). Using this approach, we detected 47 (66%) transgenic plants with mutations in the *HvCKX1* gene and 18 (64%) with mutations in the *Nud* gene (Fig. 2a, b, Table 1).

**Table 1:** Summary of genetic transformation of barley with different sgRNA constructs and PCR/RE screening of transformants for the presence of induced mutations

sgRNA construct	Edited gene	No. of explants	No. of transgenic events (%)	No. of independent transgenic plants	No. of PCR-RE detected mutants	
ckx1-sgRNA	*HvCKX1*	1143	137 (12)	71	47	
nud-sgRNA	*Nud*	1209	96 (8)	28	18	
PTG-ckx1-ckx3	*HvCKX1*	1320	142 (11)	72	49	9^a^
	*HvCKX3*				13	

^a^No. of plants with mutations in both the *HvCKX1* and *HvCKX3* genes

The disadvantage of restriction analysis is its potential inaccuracy, as some indels could be generated upstream or downstream of the diagnostic restriction site, which may lead to false negative bands on the gel. Therefore, to validate the results of the PCR/restriction analysis, we cloned and sequenced the amplicons from 25 randomly selected T_0_ plants transformed with the ckx1-sgRNA cassette. Ten clones per plant were sequenced. The mutations were detected in 22 out of 25 plants, which gave 88% efficiency (number of T_0_ plants testing positive for the mutation events). Moreover, the sequencing confirmed the presence of single nucleotide insertions or deletions, which did not disrupt the *Bsm*AI restriction enzyme site used for the initial PCR/restriction analysis (Additional file 2: Fig. S3). Interestingly, most of the tested plants appeared to be chimeric for the introduced mutations (more than two different mutations found in one T_0_ sample). Taking aside a sampling error, the T_0_ plants #34, #54, and #70 could represent the biallelic heterozygous events (only two different mutations found), while the T_0_ plant #63 could indicate the biallelic homozygous event (all mutations the same) (Additional file 2: Fig. S3). To further estimate the status of the T_0_ mutant plants, we checked the ratio between the mutated and wild-type copies of the *HvCKX1* gene in the T_0_ mutants. The wild-type copies of the *HvCKX1* gene were found in 5 out of 22 mutated plants (Table 2). The most frequent type of induced mutations was a deletion or insertion of a single nucleotide. However, larger deletions were observed in seven plants, some of them exceeding 20 bp, and the largest was the deletion of a 58 bp fragment (Additional file 2: Fig. S3).

**Table 2:** Distribution of Cas9:sgRNA induced mutations in the *HvCKX1* gene in T_0_ plants and their segregation in T_1_ progeny

T_0_ plant ID	No. of mutant and wt^a^ clones in a T_0_ plant^b^		Segregation of mutations in T_1_ lines [No. of wt, ht, and hm plants]			Segregation of T-DNA in T_1_ lines	No. of mutated, T-DNA free plants in T_1_ lines
	Mutant	wt^b^	wt	ht	hm	PCR(+):PCR(−)	
22	10	0	0	5	5	7:3	3
27	10	0	7	2	1	2:8	3
28	10	0	10	0	0	8:2	0
29	9	1	3	5	2	7:3	3
30	10	0	7	3	0	8:2	0
32	10	0	6	4	0	8:2	1
33	10	0	8	2	0	3:7	0
34	10	0	0	1	9	10:0	0
37	8	2	4	6	0	4:6	2
38	10	0	0	8	2	9:1	1
41	9	1	6	4	0	5:5	2
42	10	0	5	5	0	7:3	0
44	8	2	1	8	1	9:1	0
45	10	0	6	4	0	9:1	0
47	7	3	6	4	0	9:1	0
54	10	0	6	3	1	6:4	1
57	10	0	2	5	3	7:3	2
63	10	0	5	3	2	7:3	1
67	10	0	6	4	0	8:2	1
70	10	0	7	3	0	7:3	0
78	10	0	6	2	2	8:2	0
80	10	0	6	4	0	8:2	0
Total number of T_1_ plants:			107	85	28	156:64	20

^a^*wt* wild-type, *ht* heterozygous, *hm* homozygous

^b^Mutations detected in leaf tissue

To validate the functionality of the RNA-guided Cas9 construct for multiplex editing, we designed the PTG gene with two gRNAs targeting the *HvCKX1* and *HvCKX3* genes, respectively (Fig. 1c). The construct in the pBract211-Cas-PTG vector was used to generate transgenic barley plants by *Agrobacterium*-mediated transformation. As previously, T_0_ transformants were first screened by PCR and restriction analysis and then sequenced (Fig. 2c). The mutation frequency in the *HvCKX1* gene was comparable to that generated by a single sgRNA 49 (68%), but it was considerably lower in *HvCKX3* 13 (18%) (Table 1). Nevertheless, the overall efficiency of the PTG construct was still satisfactory, as we obtained 9 plants (21% of all mutated plants) with mutations in both CKX genes.

### Inheritance and segregation of induced mutations in the T_1_ generation

**Fig. 3:**
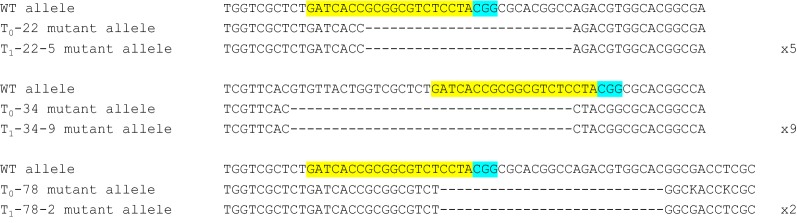
Transmission of Cas9:sgRNA induced mutations from T_0_ to T_1_ lines. Sequence alignment of wild-type *HvCKX1* and the mutant alleles identified within the same germline. The numbers of homozygous T_1_ plants with the same mutant allele are indicated on the right; the target sequence is marked in yellow and PAM motif in light blue; deletions are indicated by dashes

The inheritance and segregation of *HvCKX1* mutations was analyzed in the progeny of the 22 mutant self-pollinated T_0_ plants. Ten T_1_ progeny plants, from each T_0_ mutant plant, were screened for the presence of mutations. No mutations were found only in the progeny of the T_0_ plant #28 (Table 2). Identical mutations (long deletions) transmitted from T_0_ to T_1_ plants were found in three T_0_/T_1_ lines (i.e. #22, #34, and #78) (Fig. 3). Among 113 T_1_ mutants, eighty five were heterozygous (only one allele mutagenized), while the remaining 28 were homozygous/biallelic plants (found within 10 T_1_ lines), which gave approx. 3 homozygous plants per line. Among these 10 lines we found 26 T_1_ homozygous plants with frameshift mutations (see Additional file 3: Fig. S4). The predominant, non-Mendelian pattern of inheritance and the presence of wild-type alleles in the T_1_ generation, even from T_0_ plants originally identified as biallelic (#34, #54, #70, #63), indicated the chimeric status of T_0_ transgenic plants (Table 2). We also tested to what extent the induced mutations and T-DNA from the RNA-guided Cas9 vector segregated independently in the T_1_ generation. This allowed for discrimination of T_1_ individuals with inherited primary mutations from those in which the mutation could be potentially created de novo, because of the expression of Cas9 and sgRNA. 17.7% (20 out of 113) of T_1_ T-DNA-free individuals inherited the primary mutation in *HvCKX1* gene. T-DNA segregation in the T_1_ generations followed the Mendelian 3:1 ratio (at confidence level P = 0.99, α = 0.05) in 12 out of 22 original T_0_ events indicating a single-locus insertion of T-DNA in those plants (Table 2). The segregation ratio 9:1 or 10:0 could indicate not linked integration of two copies of T-DNA in the additional 5 T_0_ plants. 2 out of 20 T-DNA free mutants were homozygotes in terms of the mutation in the *HvCKX1* gene. This proved that it was possible to select non-transgenic mutants in the T_1_ generation by screening at least 10 plants from each T_1_ line.

### Generation of mutants with phenotypic changes

**Fig. 4:**
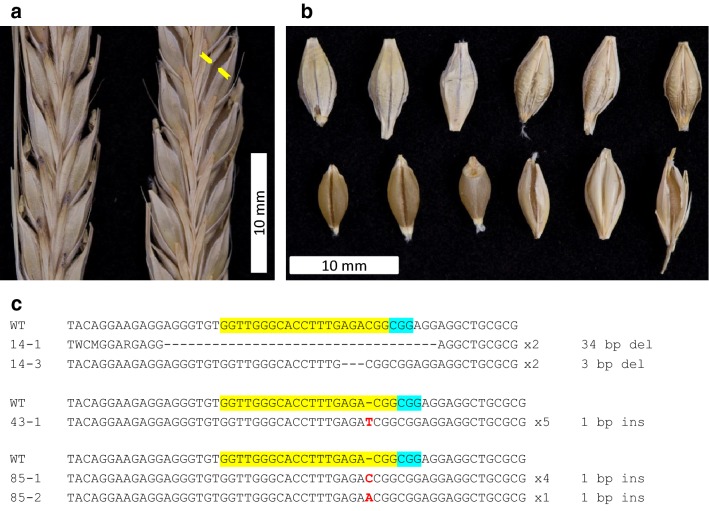
Phenotype changes generated by knock-out mutations of the *Nud* gene in barley. **a** Mature spikes of wild-type control (left) and *nud* mutant (right) plants; yellow arrowheads indicate opened hulls surrounding a naked caryopsis (all grains in the spike are naked but the hull openings are visible only at a specific angle). **b** Examples of mature covered grains from a wild-type plant (upper row) and naked grains from a *nud* mutant plant (bottom row); **c** sequence alignment of the *Nud* gene fragments cloned from T_0_ biallelic mutants. Target sequence is marked in yellow and PAM motif in light blue; deletions are indicated by dashes, and insertions by red letters

Typical domesticated barley cultivars have covered (hulled) grains with the hull firmly adhering to the caryopsis at maturity. However, there are a few cultivars, which produce naked caryopses also known as naked barley. This trait is controlled by a single locus (*nud*) on chromosome 7HL. We used a sgRNA construct targeting the *Nud* gene in the Golden Promise hulled cultivar to generate putative mutants with naked caryopses. The mutations were induced in 18 out of 25 transgenic T_0_ plants. The phenotypic changes in the form of naked caryopses with no adhering hull were observed in 11 mutants (Fig. 4a, b) which indicates for the presence of biallelic mutations in these plants. To check the mutations pattern, fragment of the *Nud* gene was sequenced in seven selected mutants. The frameshift mutations were detected in all seven plants. Moreover, two plants, #14 and #85 possessed heterozygous biallelic mutations (two different mutations), and homozygous biallelic mutation (all mutations the same) was detected only in plant #43 (Fig. 4c). Interestingly, the wild-type amplicons were detected in the remaining four mutants, which indicates for the chimeric status of these plants (see Additional file 4: Fig. S5).

## Discussion

Genome editing has become a powerful tool for functional genomics studies in plants because it allows for the precise introduction of mutations at specific genomic locations. With its outstanding efficiency, reliability, and simplicity, the RNA-guided Cas9 system quickly became the most popular technique among other available genome editing methods, however its efficiency depends on the plant species. A large number of RNA-guided Cas9 edited *Arabidopsis thaliana* plants with a mutation frequency up to 90% can be easily produced by *Agrobacterium*-infiltration of floral buds [10, 45]. In contrast, the first attempts of RNA-guided Cas9-based editing of some monocot species resulted in significantly lower mutation frequencies, such as 8.2% in rice [13], 5.6% in wheat [31], and up to 23% in barley [33]. These results indicated that the RNA-guided Cas9 system required some optimization to achieve greater mutation rates in monocots. One factor to consider is the use of appropriate binary vectors in combination with efficient protocols for stable *Agrobacterium*-mediated transformation. In this report, the pBract-based customizable binary vectors and gRNA modules were used. As targets we chose the *CKX* genes encoding the cytokinin oxidase/dehydrogenase enzymes, which permanently inactivate endogenous cytokinins. Taking into account the significance of this physiological process, we chose two *CKX* genes, *HvCKX1*and *HvCKX3*, differing in tissue specific expression patterns, of which potential knockout effects may be further studied. In contrast, the ethylene response transcription factor gene *Nud* was chosen to estimate the efficiency of the RNA-guided Cas9 system to produce visible phenotypical changes in barley, as this gene controls whether the barley grains are covered (hulled) or naked (hulles). Most of the barley cultivars including Golden Promise produce hulled grains with hulls strictly adhering to caryopses. In turn, some varieties with mutated *Nud* alleles (*nud*) produce naked (hulles) grains with non-adhering hulls. Taketa et al. [39] proved that the naked caryopsis phenotype is caused by either natural or induced mutations in the ethylene response (ERF) family transcription factor gene *Nud*. Thus, the *Nud* gene was targeted to generate mutants with naked grains.

Expression of the Cas9 gene, the use of monocot specific promoters, and an efficient transformation protocol are other factors that may affect the efficiency of the RNA-guided Cas9 system in generating knock-out, heritable mutations in barely. Strong expression of endonuclease Cas9 is desirable in order to generate a large number of double strand breaks at specific genomic locations. This can be achieved by plant codon optimization of the native Cas9 sequence in combination with monocot specific promoters. The positive relationship between codon usage and translation efficiency of heterologous proteins in plants has been established leading to, for example, 100-fold increase of cryIA protein accumulation in transgenic tobacco and tomato [46, 47]. Codon optimization has become a standard practice for heterologous proteins needed to be expressed at high levels in plants, e.g. plant-made pharmaceuticals (reviewed by [48, 49]). Some researchers indicated that use of plant codon optimized Cas9 under monocot specific promoters, such as the maize *Ubi* promoter resulted in greater mutant efficiency, which ranged from 60 to 100% in rice [7, 20], maize [26, 27], and barley (this study), as compared to the range of 8.6–45.3%, if human codon optimized Cas9 and/or the 35S promoter were used [18, 19, 33]. The expression of heterologous genes in transgenic plants can also be affected by using genetic regulatory elements such as introns. Intron mediated enhancement (IME) of gene expression in plants is a well-known phenomenon (reviewed by Laxa [50]). We used the UBQ10-i1 intron from Arabidopsis, which is among the best studied enhancing introns in plants [40]. These types of promoter-proximal introns increase the expression levels of transgenes in both dicot and monocot plants if they are positioned at the 5′ coding region [51]. The enhancing effect of the UBQ10-i1 intron has been proven in barley when used in the luciferase gene along with the *Ubi1* promoter [41]. The optimal expression of sgRNA, the second component of the RNA-guided Cas9 system, may also be important. In plants, polymerase III promoters such as U3 and U6 are frequently used for the expression of sgRNAs. In our system, the U6 promoter from wheat, which efficacy was proven in several studies [13, 31–33], has been used to drive expression of sgRNA

Our RNA-guided Cas9 system was developed based on the pBract211 vector. The pBract vectors are improved successors of the pGreen vector family, which have been widely used for transformation of cereals. The advantage of pBract vectors is their optimization for monocot plant transformation protocols. They exploit a maize ubiquitin promoter for stable and constitutive transgene expression, and contain the *hpt* gene for hygromycin selection of transformants. We have used both the pGreen and pBract vectors for transformation of even weakly susceptible cultivars of oat, wheat, and triticale [52–54]. An efficient genetic transformation method is required to produce an adequate number of edited plants with desirable mutations. For cereals, the *Agrobacterium*-mediated transformation seems to be more effective and reliable method as compared to the particle bombardment procedures [55–57]. The transformation efficiency ranging from 8 to 12% is routine for *Agrobacterium*-transformation of the barely Golden Promise cultivar in our laboratories [43]. The mutation frequency in simplex editing of the *HvCKX1* gene reached up to 88%, which is similar to the efficiency accomplished in model plants such as *Arabidopsis* [26, 45] and rice [21], and was sufficient to select homozygous mutants with frameshift mutations in the T_1_ generation. The previously reported Cas9-based mutation rates in barley obtained after *Agrobacterium*-mediated transformation ranged from 23% (23 T_0_ plants analyzed) [33] to 78% (20 T_0_ plants analyzed) [34]. Moreover, the number of biallelic mutations induced in the *Nud* gene allowed for the selection of T_0_ transformants with identifiable knockout phenotypes.

It is worth to acknowledge that also other platforms have been utilized for genome editing of barley. Meganuclease I-*Sce*I was successfully used for gene replacement by homology-directed repair [58]. TALEN technology was validated in barley by targeting phytase promoter [59] and *gfp* transgene [60, 61]. Holme et al. [62] evaluated the mature grain phytase candidate using both TALEN and RNA-guided Cas9 systems. Interestingly, the similar mutation rate of 43–44% was reported for both platforms and the induced mutations were stably inherited to the T_2_ generation. The ZFN and TALEN platforms have also been used, to a minor extent, in other important cereal species, such as rice [63–65], maize [25, 66] and wheat [31]. Although the performance of these platforms is relatively low as compared to the CRSIPR/Cas9 system, they still can be useful for specific purposes.

Simultaneous editing of multiple target sequences is desired for a more complex gene editing experimentation such as deletion of large chromosomal fragments. It is also particularly useful for knockout of multiple homologous or homeologous genes in polyploid species. As opposed to ZFN and TALENs, the RNA-guided Cas9 system is more flexible and it can be utilized for multiplex editing, since the Cas9 and sgRNAs are transcribed separately and multiple gRNA modules can be put into a single T-DNA construct. Recently, a polycistronic tRNA-gRNA system has been developed for multiplex RNA-guided Cas9-based editing in plants, which relies on endogenous mechanisms of tRNA processing [7]. In eukaryotic cells, tRNA precursors are cleaved at specific sites by RNase P and RNase Z to remove excess 5′ and 3′sequences. It has been suggested that this mechanism is used to produce tRNAs and other small RNAs such as small nucleolar RNA (snoRNA) from a single polycistronic gene. Similarly, multiple gRNAs can be assembled together with flanking tRNA sequences into a single polycistronic tRNA-gRNA gene (PTG) which requires only one U3 or U6 promoter. This greatly simplifies the RNA-guided Cas9 construct design by minimizing the sequential cloning steps needed for separate gRNAs. The effectiveness of PTG constructs in multiplex genome editing have been demonstrated in rice [7] and maize [28], where it has been proven that PTG-based system was more efficient in mutagenesis than single sgRNAs. In maize, the editing rate of tRNA-gRNA units was from 85.7 to 100% as compared to 57–71% of U6-sgRNAs in simplex editing [28]. In rice, the editing rate of tRNA-gRNA units was from 47 to 100% as compared to 44–60% of U3-sgRNAs. Moreover, up to 76% of mutations generated by tRNA-gRNA units were biallelic, whereas the U3-sgRNAs generated only 20% [7]. The similar positive effect of increased editing rate was observed in two yeast organisms, i.e. *O. polymorpha* [67] and *Y. lipolytica* [68] where the tRNA-based transcript processing was used together with Cas9 for genome editing. Another advantage of the PTG system is the fact that target sequences used in tRNA-gRNA units may start with any nucleotide, whereas sgRNA transcripts of U3 and U6 promoters are obligated to start with A and G nucleotide respectively. In this study, we designed a PTG construct for simultaneous editing of two different *CKX* genes using a tRNA sequence from barley. Our results indicate that PTG-based multiplex editing can be successfully used in barley, however, the achieved editing rate ranging from 18 to 68% was slightly lower than in rice and maize (from 47 to 100%) [7, 28]. Even though the mutation frequency in the *CKX3* gene was markedly reduced, the overall efficiency of the PTG construct was still sufficient to produce plants with mutations in both *CKX* genes.

The differences in the mutation rates between the *CKX1* (68%) and *CKX3* (18%) genes are of interest, since they are not produced by the different arrangement of the PTG construct. Presumably, they are affected by the properties of the target sequences. It has been demonstrated that the GC content of the target sequences has significant impact on the sgRNA cleavage efficiency [19, 69–72]. Therefore, the greater efficiency of *CKX1* sgRNA may result from its greater GC content (65%) as compared to *CKX3* sgRNA (55%). Moreover, both genes are located at distinct loci and show different tissue-dependent expression patterns. The highest expression level of *HvCKX1* was observed in developing grains [38], whereas *HvCKX3* in roots (data not shown). It could be possible that the highly transcribed *HvCKX1* locus is more exposed to the sgRNA/Cas9 complex as opposed to the transcriptionally inactive *HvCKX3* locus. All these factors should be taken into consideration when designing sgRNAs.

The analysis of T_1_ plants revealed that RNA-guided Cas9-induced mutations were transmitted to the next generation in at least 50% of T_1_ lines. However, only in three lines the segregation of inherited mutation coincided with Mendelian rules. The non-Mendelian segregation observed in other lines can be explained by the chimeric nature of T_0_ plants. Our results indicated that different types of mutations can be induced independently in somatic and generative tissues in T_0_ plants. Since the mutations in the T_0_ plants were detected in leaf tissue, there is no information whether these mutations were also induced in generative tissues and transmitted to gametes. This fact may explain an observation showing that wild-type T_1_ plants are a progeny of T_0_ mutants, where no wild-type clones of the *HvCKX1* gene were detected in the sampled leaf tissues (Table 2). Therefore, it is impossible to predict the segregation of mutations in T_1_ progeny based only on the analysis of somatic tissue. Based on T-DNA segregation analysis we found at least one plant with a mutation inherited independently from the Cas9-sgRNA T-DNA in 50% of T_1_ lines. The segregation of non-transgenic mutants is very important in view of commercial applications of genome editing technology in the countries where such plants are excluded from the GMO legislation.

## Conclusions

The ability to create genetic mutants is essential for the study of gene function in plants. In recent years, efforts have been made to adapt the bacterial CRISPR/Cas9 system for its application to crop plants. In this report we demonstrated the effectiveness of an optimized RNA-guided Cas9 system in the genome editing of barley. The developed set of binary vectors offers a simple, inexpensive, time-saving, and efficient way to create genome edited plants with single or multiple heritable mutations. The performed optimizations and universality of the system allows for its application to other cereal species.

## Additional files

**Additional file 1: Methods S1.** **Figure S1.** Schematic view of the pBract211 binary vector. **Figure S2.** Cloning site for the pCR8/GW/TOPO-sgRNA vector. **Table S1.** List of PCR primers and oligonucleotides used in this study. **Table S2.** List of target sequences used for genome editing in barley. **Table S3.** Composition of media for *Agrobacterium*-mediated transformation and in vitro regeneration of barley immature embryos.

**Additional file 2: Figure S3.** Sequence alignment of the *HvCKX1* gene fragments cloned from selected T_0_ plants. Target sequence is marked in yellow and PAM motif in light blue; deletions are indicated by dashes.

**Additional file 3: Figure S4.** Sequence alignment of the *HvCKX1* gene fragments cloned from selected T_1_ plants. Target sequence is marked in yellow and PAM motif in light blue; deletions are indicated by dashes.

**Additional file 4: Figure S5.** Sequence alignment of the *Nud* gene fragments cloned from selected T_0_ plants. Target sequence is marked in yellow and PAM motif in light blue; deletions are indicated by dashes.
