# Supplementary material for: A simple and efficient CRISPR/Cas9 platform for induction of single and multiple, heritable mutations in barley (Hordeum vulgare L.)
Source: Plant Methods. 2018 Dec 18;14:111. doi: 10.1186/s13007-018-0382-8 (PMC6297969; doi:10.1186/s13007-018-0382-8)
Supplement: Supplementary file 1 — Additional file 1: Methods S1. Figure S1. Schematic view of the pBract211 binary vector. Figure S2. Cloning site for the pCR8/GW/TOPO-sgRNA vector. Table S1. List of PCR primers and oligonucleotides used in this study. Table S2. List of target sequences used for genome editing in barley. Table S3. Composition of media for Agrobacterium-mediated transformation and in vitro regeneration of barley immature embryos. [file 13007_2018_382_MOESM1_ESM.docx]

**Methods S1**

Cloning of the pBract211-Cas-GW vector

The pBrct211 vector (Fig. S1) and Cas9 sequence were double digested with the *Xma*I and *Spe*I restriction enzymes. The Cas9 insert was then ligated with linearized pBract211 vector using T4 DNA ligase (NEB). After ligation, the vector was electroporated into the *E. coli* strain DH5α and spread on agar plates for selection of positive colonies. Next, the pBract211-Cas9 vector was isolated from a single positive colony and cut by the *Sph*I enzyme. The sticky ends generated by *Sph*I were blunted using T4 DNA polymerase. The linearized vector was dephosphorylated and ligated with the Gateway cassette from the Gateway conversion Kit (Invitrogen). The resulting Gateway destination vector pBract211-Cas9-GW was later used for Gateway cloning of the U6-sgRNA cassette.

**Fig. S1** Schematic view of the pBract211 binary vector.

Cloning of the pCR8/GW/TOPO-sgRNA vector

The DNA fragment consisting of the wheat U6 RNA promoter, *Bsa*I cloning site insert, and gRNA scaffold (Fig. 1) were synthesized and cloned into the pCR8/GW/TOPO Gateway entry vector (Invitrogen). After cutting the vector with the *Bsa*I enzyme, the forward 5’-CTTG(N)_20_ and reverse 5’-AAAC(N)_20_-3’ complementary target sequences are cloned in the form of an annealed, double stranded oligo DNA with 5’ and 3’ 4nt overhangs complementary to the vector backbone overhangs generated by *Bsa*I. The following procedure was used for oligo annealing and ligation:

| Annealing | | Reaction conditions |
| --- | --- | --- |
| 100 µM forward oligo | 1µl | Incubation in thermal cycler: step 1 – 95°C/5 min, step 2 – decrease temp. by 0.2°C/s to 70°C, step 3 – decrease temp. by 0.1°C/s to 25°C |
| 100 µM reverse complement oligo | 1µl |  |
| T4 ligase buffer 10× (NEB) | 1µl |  |
| H_2_O | 7µl |  |
| Ligation | | Reaction conditions |
| annealed oligos diluted 200× | ≈ 0.15 pmol ends | Incubation at 22°C for 10 min. |
| Vector 50 ng/µl | ≈ 0.05 pmol ends |  |
| Quick ligation buffer 2x (NEB) | 5 µl |  |
| T4 ligase (NEB) | 1 µl |  |
| H_2_O | to 10 µl |  |

After incubation, 1µl of ligation mixture was taken directly for electroporation of *E. coli* strain DH5α. The sequence of the U6sgRNA cassette was verified by sequencing.


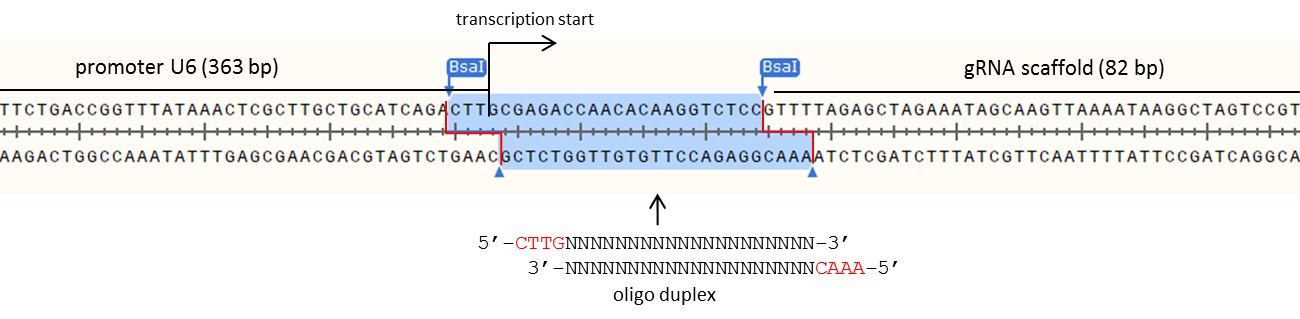


**Fig. S2** Cloning site for the pCR8/GW/TOPO-sgRNA vector. After cutting with *Bsa*I, the sequence shaded in blue is replaced by the designed oligo duplex using complementary overhangs (indicated in red).

Assembly of the PTG unit (polycistronic tRNA-gRNA)

For multiplex editing we used a synthesized PTG fragment consisting of two spacers for the *HvCKX1* and *HvCKX3* genes, respectively (Table S2). This fragment was cut with *Bsa*I and cloned into the pCR8/GW/TOPO-U6-sgRNA vector by ligation of the *Bsa*I overhangs. It is possible to use this fragment for assembly of the custom PTG cassette with any spacer sequences by performing an overlapping extension PCR. The following example shows the PCR primers which can be used for assembly of the PTG fragment with 3 target sequences (*Bsa*I sites are underlined):

1^st^ PCR reaction: fragment1

primer T1F 5’-ggtctcccttgcaacaaagcgca-3’

primer T1R 5’-NNNNNNNNNNNNNNNNNNNNtgcgcatccagggaatcgaac-3’

spacer1 reverse complement

2^nd^ PCR reaction: fragment 2

spacer1 forward

primer G2F 5’-NNNNNNNNNNNNNNNNNNNNgttttagagctagaaatagcaag-3’

primer T2r 5’-NNNNNNNNNNNNNNNNNNNNtgcgcatccagggaatc-3’

spacer 2 reverse complement

3^rd^ PCR reaction: fragment 3

spacer 2 forward

primer G3F 5’-NNNNNNNNNNNNNNNNNNNNgttttagagctagaaatagcaag-3’

primer T3r 5’-ggtctcgaaacNNNNNNNNNNNNNNNNNNNNtgcgcatccagggaatc-3’ spacer 3 reverse complement

4^th^ PCR reaction: assembly of the PTG fragment with *Bsa*I ends

Take 1µl of fragments 1, 2, and 3 from the reaction mixtures. Use primers T1F/T3R for the PCR reaction. Run the PCR product on an agarose gel and excise the appropriate sized fragment. Use a spin column kit to elute the band from the gel. After cleanup, clone the PTG fragment into a plasmid vector of your choice. Confirm the sequence by Sanger sequencing using vector specific primers. If the sequence is correct, excise the verified PTG fragment from the plasmid by cutting with the *Bsa*I enzyme, and clone it into the U6-sgRNA cassette in the your Gateway entry vector

**Table S1**. List of PCR primers and oligonucleotides used in this study.

| purpose | name | sequence |
| --- | --- | --- |
| oligos for cloning of HvCKX1 sgRNA | ckx1-sg1A | 5’-CTTGatcaccgcggcgtctccta-3’ |
|  | ckx1-sg1B | 5’-AAACtaggagacgccgcggtgat-3’ |
| oligos for cloning of *Nud* sgRNA | nud-sg1A | 5’-CTTGttgggcacctttgagacgg-3’ |
|  | nud-sg1B | 5’-AAACccgtctcaaaggtgcccaa-3’ |
| PCR primers flanking the *HvCKX1* target sequence | ckx1-psF | 5’-CAGCTTTCACCGTAGCAGCA-3’ |
|  | ckx1-psR | 5’-AACGAGATGGTGTACGGCCA-3’ |
| PCR primers flanking the *HvCKX3* target sequence | ckx3-psF | 5’-TCTCTCTCTCGCCCTTTGCT-3’ |
|  | ckx3-psR | 5’-CATCTCCACCACAATGCCAT-3’ |
| PCR primers flanking the *Nud* target sequence | nud-psF | 5’-TCCAAGAAGAAGTTTCGCGG-3’ |
|  | nud-psR | 5’-ATGATCTCCCCGTTGGCACT-3’ |
| Amplification of the TaU6sgRNA cassette | TaU6-F | 5’-GACCAAGCCCGTTATTCTGA-3’ |
|  | sg-R | 5’-AAAAAAAGCACCGACTCGGTGCCAC-3’ |

**Table S2**. List of target sequences used for genome editing in barley

| Gene | target sequence | sgRNA sequence / PTG sequence |
| --- | --- | --- |
| HvCKX1 | gatcaccgcggcgtctcctacgg | gatcaccgcggcgtctcctagttttagagctagaaatagcaagttaaaataaggctagtccgttatcaacttgaaaaagtggcaccgagtcggtgcttttttt |
| Nud | ggttgggcacctttgagacggcgg | ggttgggcacctttgagacggttttagagctagaaatagcaagttaaaataaggctagtccgttatcaacttgaaaaagtggcaccgagtcggtgcttttttt |
| HvCKX1 (for PTG cassette) | gatcaccgcggcgtctcctacgg | caacaaagcgcatctggtgtagtggtatcatagtaccctcccacggtactgaccagggttcgattccctggatgcgcagatcaccgcggcgtctcctagttttagagctagaaatagcaagttaaaataaggctagtccgttatcaacttgaaaaagtggcaccgagtcggtgccaacaaagcgcatctggtgtagtggtatcatagtaccctcccacggtactgaccagggttcgattccctggatgcgcagcaagttcatccagagccccagttttagagctagaaatagcaagttaaaataaggctagtccgttatcaacttgaaaaagtggcaccgagtcggtgcttttttt |
| HvCKX3 (for PTG cassette) | Gcaagttcatccagagccccatgg |  |

**Table S3**. Composition of media for *Agrobacteriu*-transformation and in vitro regeneration of barley immature embryos.

| **components** | **medium** | | | |
| --- | --- | --- | --- | --- |
|  | **CI** | **TR** | **Reg** | **half-strength MS** |
| MS basal salts (Duchefa M0221) | 4.3 g/L | n/a | n/a | 2.15 g/L |
| MS salts NH_4_ free (Duchefa M0238) | n/a | 2.7 g/L | 2.7 g/L | n/a |
| NH_4_NO_3_ | n/a | 165 mg/L | 165 mg/L | n/a |
| CuSO_4_ · 5H_2_O | 1.25 mg/L | n/a | n/a | n/a |
|  |  |  |  |  |
| Casein hydrolysate | 1 g/L | n/a | n/a | n/a |
| Maltose | 30 g/L | 20 g/L | 20 g/L | 20 g/L |
| Glutamine | n/a | 750 mg/L | 750 mg/L | n/a |
|  |  |  |  |  |
| Myo-inositol | 350 mg/L | 100 mg/L | 100 mg/L | n/a |
| Proline | 690 mg/L | n/a | n/a | n/a |
| Thiamine · HCl | 1 mg/L | 0.4 mg/L | 0.4 mg/L | n/a |
|  |  |  |  |  |
| 2,4-D | n/a | 2.5 mg/L | n/a | n/a |
| BAP | n/a | 1 mg/L | n/a | n/a |
| DICAMBA | 2.5 mg/L | n/a | n/a | n/a |
|  |  |  |  |  |
| Higromycin B | 50 mg/L^1)^ | 50 mg/L | 50 mg/L | 50 mg/L |
| Timentin | 150 mg/L | 150 mg/L | 150 mg/L | 150 mg/L |
|  |  |  |  |  |
| Gelrite (Duchefa G1101) | 3 g/L | 3 g/L | 3 g/L | 3 g/L |

^1)^ No hygromycin is added to CI medium for co-cultivation with *A. tumefaciens*

DNA sequence of Cas9; the sequence of UBQ10 1st intron is shaded:

5’-CCCGGGATCCGCCATGGCCCCGAAGAAGAAGAGGAAGGTGGGCATGGACAAGAAGTACTCCATCGGCCTGGACATCGGGACCAACTCTGTAGGCTGGGCGGTGATCACGGACGAGTATAAGGTGCCCTCGAAGAAGTTCAAGGTCCTGGGAAACACCGACCGTCACAGCATCAAGAAGAACCTGATAGGGGCCCTCCTGTTCGACTCAGGTAAATTTCTGTGTTCCTTATTCTCTCAAAATCTTCGATTTTGTTTTCGTTCGATCCCAATTTCGTATATGTTCTTTGGTTTAGATTCTGTTAATCTTAGATCGAAGACGATTTTCTGGGTTTGATCGTTAGATATCATCTTAATTCTCGATTAGGGTTTCATAGATATCATCCGATTTGTTCAAATAATTTGAGTTTTGTCGAATAATTACTCTTCGATTTGTGATTTCTATCTAGATCTGGTGTTAGTTTCTAGTTTGTGCGATCGAATTTGTCGATTAATCTGAGTTTTTCTGATTAACAGGTGAGACTGCTGAGGCGACCAGGCTGAAGCGTACTGCAAGAAGACGTTACACGAGGCGGAAGAACAGGATCTGCTACCTCCAAGAGATCTTCAGCAACGAGATGGCGAAGGTCGACGACTCGTTCTTCCACCGTCTCGAGGAGAGCTTCCTGGTCGAGGAAGACAAGAAGCACGAGAGGCACCCGATCTTCGGGAACATCGTGGACGAAGTGGCGTACCACGAGAAGTACCCCACAATCTACCACCTCCGGAAGAAGCTGGTTGACTCCACTGACAAGGCTGACCTGCGTCTCATCTACCTGGCCCTCGCCCACATGATCAAGTTCAGGGGTCACTTCCTCATAGAGGGGGACCTGAACCCTGACAATAGCGACGTCGACAAGCTGTTCATCCAGCTGGTGCAAACCTACAACCAGCTCTTCGAGGAGAACCCCATCAACGCTAGTGGAGTTGACGCCAAGGCTATCCTGTCTGCTAGGCTGTCCAAAAGCCGACGCTTGGAGAACTTAATCGCTCAGCTTCCGGGGGAGAAGAAGAACGGACTCTTCGGGAACCTGATTGCCCTGAGTCTGGGCCTGACCCCGAACTTCAAAAGCAACTTCGACCTCGCCGAGGACGCGAAACTCCAGCTGTCAAAGGACACATACGACGACGACCTGGACAACCTGTTAGCACAGATCGGGGACCAGTACGCCGACCTCTTTCTGGCAGCTAAGAACCTGTCTGACGCCATCCTCCTGTCCGACATCCTCCGAGTCAACACTGAGATCACCAAGGCCCCACTGAGCGCCTCTATGATAAAACGCTACGACGAGCACCACCAGGACCTGACTCTCCTGAAGGCTCTCGTACGTCAGCAGCTGCCTGAGAAGTACAAGGAGATCTTCTTCGACCAGTCGAAGAACGGCTACGCCGGGTACATTGATGGCGGGGCTTCTCAAGAGGAGTTCTACAAGTTCATCAAGCCCATCCTCGAGAAGATGGACGGCACCGAGGAGCTGCTCGTTAAGTTGAACAGAGAGGACCTGCTCCGAAAGCAGAGGACCTTTGATAACGGGTCGATCCCGCACCAGATCCATCTCGGTGAGCT**C**CATGCTATCCTGCGACGCCAAGAGGACTTCTACCCCTTCCTCAAGGACAACCGCGAGAAGATTGAGAAGATCCTGACATTCCGAATCCCGTACTACGTCGGGCCTCTTGCAAGGGGCAACAGCCGGTTCGCTTGGATGACCCGCAAGTCGGAGGAGACTATCACACCCTGGAACTTTGAGGAGGTCGTGGACAAGGGCGCATCTGCCCAGAGTTTCATCGAGAGGATGACCAACTTCGATAAGAACCTCCCGAACGAGAAGGTGCTCCCGAAGCACTCTCTGCTCTACGAGTACTTCACCGTGTACAACGAGCTGACGAAGGTCAAGTACGTGACCGAGGGTATGCGGAAGCCTGCGTTCCTGAGCGGGGAGCAAAAGAAGGCCATCGTCGATCTCCTGTTCAAGACCAACCGCAAGGTGACCGTCAAGCAGCTCAAGGAGGACTACTTCAAGAAGATCGAGTGCTTCGACTCCGTCGAGATCTCGGGCGTTGAGGACAGGTTCAACGCAAGCCTGGGGACCTACCATGACCTGCTCAAGATTATCAAGGACAAGGACTTCCTCGACAACGAGGAGAATGAGGACATTCTAGAGGACATCGTGCTCACGCTGACCCTCTTCGAGGACCGCGAGATGATCGAGGAGCGCCTCAAGACCTACGCCCACCTCTTCGACGACAAGGTGATGAAGCAACTGAAGAGGCGCAGGTATACTGGATGGGGGAGGCTCTCAAGGAAGCTGATCAACGGCATCAGGGACAAGCAGAGCGGCAAGACGATCCTCGACTTCCTGAAGTCTGACGGCTTCGCTAACCGCAACTTCATGCAGCTCATCCACGACGACTCGCTCACGTTCAAGGAAGACATCCAGAAGGCGCAGGTAAGCGGCCAGGGTGATTCTCTGCATGAGCATATCGCTAACCTCGCCGGCTCCCCCGCAATCAAAAAGGGAATCCTCCAGACCGTCAAGGTCGTGGACGAGCTGGTTAAAGTGATGGGCAGGCACAAGCCTGAGAACATCGTGATCGAGATGGCGCGCGAGAATCAGACGACTCAGAAGGGCCAGAAGAACTCGCGCGAGCGCATGAAGAGAATTGAGGAGGGTATCAAGGAACTCGGCAGCCAGATCCTCAAGGAGCACCCAGTTGAGAACACCCAGCTCCAGAATGAGAAGCTGTACCTCTACTACCTGCAGAACGGCAGGGACATGTACGTGGACCAAGAGCTGGACATCAACAGGCTGTCCGACTACGACGTTGACCATATCGTGCCCCAAAGTTTCCTCAAGGACGACTCGATCGACAACAAGGTGCTCACGAGGTCCGACAAGAACCGCGGCAAGTCTGACAACGTGCCAAGCGAGGAGGTCGTGAAGAAGATGAAGAACTACTGGCGGCAGCTCCTGAACGCCAAGCTCATCACCCAGCGCAAGTTCGACAACTTGACAAAGGCAGAGAGAGGAGGCCTGTCGGAGCTTGATAAGGCGGGCTTCATTAAGAGGCAGCTCGTCGAGACTAGGCAGATCACGAAGCACGTGGCGCAGATCCTGGATAGCAGGATGAACACGAAGTACGACGAGAACGACAAGCTCATCCGCGAGGTGAAGGTCATCACCCTCAAGTCCAAGCTGGTGAGCGACTTCCGCAAGGATTTCCAGTTCTACAAGGTCCGCGAGATCAACAACTACCACCACGCCCACGACGCCTACCTGAACGCGGTAGTTGGCACGGCCCTCATCAAGAAGTACCCTAAGCTCGAGAGCGAGTTCGTGTACGGCGACTACAAGGTGTACGACGTGCGCAAGATGATCGCGAAGTCGGAGCAGGAGATCGGCAAGGCGACCGCCAAGTACTTCTTCTACAGCAACATCATGAACTTCTTCAAGACCGAGATCACGCTGGCCAACGGCGAGATCAGAAAGAGGCCGCTCATTGAGACTAATGGTGAGACTGGCGAGATCGTGTGGGATAAGGGAAGGGACTTCGCGACTGTGCGCAAGGTTCTGAG**T**ATGCCTCAGGTGAACATCGTCAAGAAGACCGAGGTCCAGACCGGCGGCTTCAGCAAGGAGTCAATCCTCCCGAAGAGGAACAGCGATAAGCTGATTGCGAGGAAGAAGGACTGGGACCCAAAGAAGTACGGCGGGTTCGATAGCCCAACCGTGGCTTACAGTGTGCTGGTGGTGGCGAAGGTCGAGAAGGGTAAGTCCAAGAAGCTCAAGTCCGTGAAGGAGCTGCTCGGCATCACCATCATGGAGCGGTCCAGCTTCGAGAAGAACCCAATCGACTTCCTCGAGGCCAAGGGCTATAAGGAGGTCAAGAAGGACCTCATCATCAAGCTGCCAAAGTACTCCCTGTTCGAGCTTGAGAACGGCCGCAAGAGGATGCTCGCTTCTGCAGGAGAGTTGCAGAAAGGCAACGAGCTTGCGCTCCCGTCCAAGTACGTCAACTTCCTGTACCTCGCCAGCCACTACGAGAAGCTGAAGGGCTCTCCAGAGGACAATGAGCAGAAGCAGCTATTCGTCGAGCAGCACAAGCACTACCTGGACGAGATCATCGAGCAGATCAGCGAGTTCAGCAAGAGGGTGATCCTCGCCGACGCGAACCTGGATAAGGTGCTCTCCGCCTACAACAAGCACCGCGACAAGCCAATTCGCGAACAGGCCGAAAACATTATACACCTGTTTACGCTCACAAACCTGGGTGCGCCAGCAGCCTTCAAGTACTTCGATACCACCATCGACCGCAAGCGGTACACCTCCACTAAGGAGGTGCTCGACGCGACCCTGATCCACCAGTCTATCACCGGTCTCTACGAAACAAGGATCGACCTGTCTCAGCTCGGAGGTGATTGAGCTCACTAGT-3’

DNA sequence of TaU6sgRNA cassette; sequence of the U6 promoter is in blue, restriction sites for BsaI are underlined and the sequence of gRNA scaffold is in red:

5’-gaccaagcccgttattctgacagttctggtgctcaacacatttatatttatcaaggagcacattgttactcactgctaggagggaatcgaactaggaatattgatcagaggaactacgagagagctgaagataactgccctctagctctcactgatctgggtcgcatagtgagatgcagcccacgtgagttcagcaacggtctagcgctgggcttttaggcccgcatgatcgggcttttgtcgggtggtcgacgtgttcacgattggggagagcaacgcagcagttcctcttagtttagtcccacctcgcctgtccagcagagttctgaccggtttataaactcgcttgctgcatcagacttgc**gagacc**aacacaa**ggtctc**cgttttagagctagaaatagcaagttaaaataaggctagtccgttatcaacttgaaaaagtggcaccgagtcggtgcttttttt-3’
