# Supplementary material for: A simple and efficient CRISPR/Cas9 platform for induction of single and multiple, heritable mutations in barley (Hordeum vulgare L.)
Source: Plant Methods. 2018 Dec 18;14:111. doi: 10.1186/s13007-018-0382-8 (PMC6297969; doi:10.1186/s13007-018-0382-8)
Supplement: Supplementary file 2 — Additional file 2: Figure S3. Sequence alignment of the HvCKX1 gene fragments cloned from selected T0 plants. Target sequence is marked in yellow and PAM motif in light blue; deletions are indicated by dashes. [file 13007_2018_382_MOESM2_ESM.pdf]

Supplementary figures

Fig. S3

```
WT      TCGTTCACGTGTTACTGGTCGCTCTGATCACCGCGGCGTCTC-CTACGGCGCACGGCCAGACGTGGCACGGCGACCTCGC        279
22-10   TCGTTCACGTGTTACTGGTCGCTCTGATCACCGCGGCGTCTCACTACGGCGCACGGCCAGACGTGGCACGGCGACCTCGC        253
22-1    TCGTTCACGTGTTACTGGTCGCTCTGATCACCGCGGCGTCTCTCTACGGCGCACGGCCAGACGTGGCACGGCGACCTCGC        254
22-2    TCGTTCACGTGTTACTGGTCGCTCTGATCACC--------------------------AGACGTGGCACGGCGACCTCGC        227
22-3    TCGTTCACGTGTTACTGGTCGCTCTGATCACC--------------------------AGACGTGGCACGGCGACCTCGC        230
22-4    TCGTTCACGTGTTACTGGTCGCTCTGATCACCGCGGCGTCTCGCTACGGCGCACGGCCAGACGTGGCACGGCGACCTCGC        252
22-5    TCGTTCACGTGTTACTGGTCGCTCTGATCACC--------------------------AGACGTGGCACGGCGACCTCGC        228
22-6    TCGTTCACGTGTTACTGGTCGCTCTGATCACC--------------------------AGACGTGGCACGGCGACCTCGC        230
22-7    TCGTTCACGTGTTACTGGTCGCTCTGATCACC--------------------------ASACGTGGCACGGCGACCTCGC        228
22-8    TCGTTCACGTGTTACTGGTCGCTCTGATCACC--------------------------AGACGTGGCACGGCGACCTCGC        228
22-9    TCGTTCACGTGTTACTGGTCGCTCTGATCACCGCGGCGTCTCACTACGGCGCACGGCCAGACGTGGCACGGCGACCTCGC        256

WT      TCGTTCACGTGTTACTGGTCGCTCTGATCACCGCGGCGTCTC-CTACGGCGCA-CGGCCAGACGTGGCACGGCGACCTCG        278
27-10   TCGTTCACGTGTTACTGGTCGCTCTGATCACCGCGGCGTCTCTCTACGGCGCA-CGGCCAGACGTGGCACGGCGACCTCG        253
27-1    TCGTTCACGTGTTACTGGTCGCTCTGATCACCGCGGCGTCTCACTACGGCGCA-CGGCCAGACGTGGCACGGCGACCTCG        253
27-2    TCGTTCACGTGTTACTGGTCGCTCTGATCACCGCGGCGTCTCCCTACGGCGCA-CGGCCAGACGTGGCACGGCGACCTCG        254
27-4    TCGTTCACGTGTTACTGGTCGCTCTGATCACCGCGGCGTCTCACTACGGCGCA-CGGCCAGACGTGGCACGGCGACCTCG        253
27-5    TCGTTCACGTGTTACTGGTCGCTCTGATCACCGCGGTACGCCGCCGTGCCACGGCGACCAGACGTGGCACGGCGACCTCG        256
27-6    TCGTTCACGTGTTACTGGTCGCTCTGATCACCGCGGCGTCTCCCTACGGCGCA-CGGCCAGACGTGGCACGGCGACCTCG        252
27-7    TCGTTCACGTGTTACTGGTCGCTCTGATCACCGCGGCGTCTCTCTACGGCGCA-CGGCCAGACGTGGCACGGCGACCTCG        254
27-8    TCGTTCACGTGTTACTGGTCGCTCTGATCACCGCGGCGTCTCTCTACGGCGCA-CGGCCAGACGTGGCACGGCGACCTCG        252
27-9    TCGTTCACGTGTTACTGGTCGCTCTGATCACCGCGGCGTCTCACTACGGCGCA-CGGCCAGACGTGGCACGGCGACCTCG        253
27-3    TCGTTCACGTGTTACTGGTCGCTCTGATCACCGCGGCGTCTCACTACGGCGCA-CGGCCAGACGTGGCACGGCGACCTCG        246

WT      TCGTTCACGTGTTACTGGTCGCTCTGATCACCGCGGCGTCTCC--TACGGCGCACGGCCAGACGTGGCACGGCGACCTCGCGGCGCTCGCCGCGGCAGGCAAGCTCCGCAGCGACCCCAA        318
28-6    TCGTTCACGTGTTACTGGTCGCTCTGATCACCGCGGCGTCTCTC-TACGGCGCACGGCCAGACGTGGCACGGCGACCTCGCGGCGCTCGCCGCGGCAGGCAAGCTCCGCAGCGACCCCAA        294
28-7    TCGTTCACGTGTTACTGGTCGCTCTGATCACCGCGGCGTCTCTC-TACGGCGCACGGCCAGACGTGGCACGGCGACCTCGCGGCGCTCGCCGCGGCAGGCAAGCTCCGCAGCGACCCCAA        294
28-8    TCGTTCACGTGTTACTGGTCGCTCTGATCACCGCGGCGTCTCCCCTACGGCGCACGGCCAGACGTGGCACGGCGACCTCGCGGCGCTCGCCGCGGCAGGCAAGCTCCGCAGCGACCCCAA        293
28-9    TCGTTCACGTGTTACTGGTCGCTCTGATCACCGCGGCGTCTCTC-TACGGCGCACGGCCAGACGTGGCACGGCGACCTCGCGGCGCTCGCCGCGGCAGGCAAGCTCCGCAGCGACCCCAA        293
28-10   TCGTTCACGTGTTACTGGTCGCA----------------------------------------------------------GGCGCTCGCCGCGGCAGGCAAGCTCCGCAGCGACCCCAA        235
28-2    TCGTTCACGTGTTACTGGTCGCA----------------------------------------------------------GGCGCTCGCCGCGGCAGGCAAGCTCCGCAGCGACCCCAA        237
28-1    TCGTTCACGTGTTACTGGTCGCTCTGATCACCGCGGCGTCTCCC-TACGGCGCACGGCCAGACGTGGCACGGCGACCTCGCGGCGCTCGCCGCGGCAGGCAAGCTCCGCAGCGACCCCAA        294
28-3    TCGTTCACGTGTTACTGGTCGCTCTGATCACCGCGGCGTCTCTC-TACGGCGCACGGCCAGACGTGGCACGGCGACCTCGCGGCGCTCGCCGCGGCAGGCAAGCTCCGCAGCGACCCCAA        293
28-4    TCGTTCACGTGTTACTGGTCGCTCTGATCACCGCGGCGTCTCAC-TACGGCGCACGGCCAGACGTGGCACGGCGACCTCGCGGCGCTCGCCGCGGCAGGCAAGCTCCGCAGCGACCCCAA        292
28-5    TCGTTCACGTGTTACTGGTCGCTCTGATCACCGCGGCGTCTCAC-TACGGCGCACGGCCAGACGTGGCACGGCGACCTCGCGGCGCTCGCCGCGGCAGGCAAGCTCCGCAGCGACCCCAA        293
```

```
WT      ATCGTTCACGTGTTACTGGTCGCTCTGATCACCGCGGCGTCTC-CTACGGCGCACGGCCAGACGTGGCACGGCGACCTCG     278
29-1    ATCGTTCACGTGTTACTGGTCGCTCTGATCACCGCGGCGTCTC--TACGGCGCACGGCCAGACGTGGCACGGCGACCTCG     252
29-10   ATCGTTCACGTGTTACTGGTCGCTCTGATCACCGCGGCGTCTCACTACGGCGCACGGCCAGACGTGGCACGGCGACCTCG     252
29-2    ATCGTTCACGTGTTACTGGTCGCTCTGATCACCGCGGCGTCTC--TACGGCGCACGGCCAGACGTGGCACGGCGACCTCG     252
29-3    ATCGTTCACGTGTTACTGGTCGCTCTGATCACCGCGGCGTCTCACTACGGCGCACGGCCAGACGTGGCACGGCGACCTCG     252
29-4    ATCGTTCACGTGTTACTGGTCGCTCTGATCACCGCGGCGTCTC-CTACGGCGCACGGCCAGACGTGGCACGGCGACCTCG     253
29-5    ATCGTTCACGTGTTACTGGTCGCTCTGATCACCGCGGCGTCTC--TACGGCGCACGGCCAGACGTGGCACGGCGACCTCG     253
29-6    ATCGTTCACGTGTTACTGGTCGCTCTGATCACCGCGGCGTCTCCCTACGGCGCACGGCCAGACGTGGCACGGCGACCTCG     255
29-7    ATCGTTCACGTGTTACTGGTCGCTCTGATCACCGCGGCGTCTCCCTACGGCGCACGGCCAGACGTGGCACGGCGACCTCG     254
29-8    ATCGTTCACGTGTTACTGGTCGCTCTGATCACCGCGGCGTCTCTCTACGGCGCACGGCCAGACGTGGCACGGCGACCTCG     252
29-9    ATCGTTCACGTGTTACTGGTCGCTCTGATCACCGCGGCGTCTCCCTACGGCGCACGGCCAGACGTGGCACGGCGACCTCG     252

WT      TCGTTCACGTGTTACTGGTCGCTCTGATCACCGCGGCGTCTC-CTACGGCGCACGGCCAGACGTGGCACGGCGACCTCGC     279
30-10   TCGTTCACGTGTTACTGGTCGCTCTGATCACCGCGGCGTCTCACTACGGCGCACGGCCAGACGTGGCACGGCGACCTCGC     253
30-1    TCGTTCACGTGTTACTGGTCGCTCTGATCACCGCGGCGTCTCACTACGGCGCACGGCCAGACGTGGCACGGCGACCTCGC     254
30-2    TCGTTCACGTGTTACTGGTCGCTCTGATCACCGCGGCGTCTCACTACGGCGCACGGCCAGACGTGGCACGGCGACCTCGC     253
30-3    TCGTTCACGTGTTACTGGTCGCTCTGATCACCGCGGCGTCTCACTACGGCGCACGGCCAGACGTGGCACGGCGACCTCGC     256
30-4    TCGTTCACGTGTTACTGGTCGCTCTGATCACCGCGGCGTCTCACTACGGCGCACGGCCAGACGTGGCACGGCGACCTCGC     253
30-5    TCGTTCACGTGTTACTGGTCGCTCTGATCACCGCGGCGTCTC--TACGGCGCACGGCCAGACGTGGCACGGCGACCTCGC     252
30-6    TCGTTCACGTGTTACTGGTCGCTCTGATCACCGCGGCGTCT--CTACGGCGCACGGCCAGACGTGGCACGGCGACCTCGC     253
30-7    TCGTTCACGTGTTACTGGTCGCTCTGATCACCGCGGCGTCTCCCTACGGCGCACGGCCAGACGTGGCACGGCGACCTCGC     255
30-8    TCGTTCACGTGTTACTGGTCGCTCTGATCACCGCGGCGTCTCCCTACGGCGCACGGCCAGACGTGGCACGGCGACCTCGC     253
30-9    TCGTTCACGTGTTACTGGTCGCTCTGATCACCGCGGCGTCT--CTACGGCGCACGGCCAGACGTGGCACGGCGACCTCGC     253

WT      TCGTTCACGTGTTACTGGTCGCTCTGATCACCGCGGCGTCTCC-TACGGCGCACGGCCAGACGTGGCACGGCGACCTCGC     279
32-10   TCGTTCACGTGTTACTGGTCGCTCTGATCACCGCGGCGTCTC--TACGGCGCACGGCCAGACGTGGCACGGCGACCTCGC     251
32-1    TCGTTCACGTGTTACTGGTCGCTCTGATCACCGCGGCGTCTC--TACGGCGCACGGCCAGACGTGGCACGGCGACCTCGC     251
32-2    TCGTTCACGTGTTACTGGTCGCTCTGATCACCGCGGCGTCTCACTACGGCGCACGGCCAGACGTGGCACGGCGACCTCGC     253
32-3    TCGTTCACGTGTTACTGGTCGCTCTGATCACCGCGGCGTCTCT--ACGGCGCACGGCCAGACGTGGCACGGCGACCTCGC     252
32-4    TCGTTCACGTGTTACTGGTCGCTCTGATCACCGCGGCGTCTC--TACGGCGCACGGCCAGACGTGGCACGGCGACCTCGC     251
32-5    TCGTTCACGTGTTACTGGTCGCTCTGATCACCGCGGCGTCTC--TACGGCGCACGGCCAGACGTGGCACGGCGACCTCGC     251
32-6    TCGTTCACGTGTTACTGGTCGCTCTGATCACCGCGGCGTCTC--TACGGCGCACGGCCAGACGTGGCACGGCGACCTCGC     251
32-7    TCGTTCACGTGTTACTGGTCGCTCTGATCACCGCGGCGTCTCTCTACGGCGCACGGCCAGACGTGGCACGGCGACCTCGC     254
32-8    TCGTTCACGTGTTACTGGTCGCTCTGATCACCGCGGCGTCTC--TACGGCGCACGGCCAGACGTGGCACGGCGACCTCGC     252
32-9    TCGTTCACGTGTTACTGGTCGCTCTGATCACCGCGGCGTCTCT--ACGGCGCACGGCCAGACGTGGCACGGCGACCTCGC     251

WT      TCGTTCACGTGTTACTGGTCGCTCTGATCACCGCGGCGTCTC-CTACGGCGCACGGCCAGACGTGGCACGGCGACCTCGC     279
33-10   TCGTTCACGTGTTACTGGTCGCTCTGATCACCGCGGCGTCTCTCTACGGCGCACGGCCAGACGTGGCACGGCGACCTCGC     253
33-1    TCGTTCACGTGTTACTGGTCGCTCTGATCACCGCGGCGTCTCCCTACGGCGCACGGCCAGACGTGGCACGGCGACCTCGC     254
33-2    TCGTTCACGTGTTACTGGTCGCTCTGATCACCGCGGCGTCTCTCTACGGCGCACGGCCAGACGTGGCACGGCGACCTCGC     252
33-3    TCGTTCACGTGTTACTGGTCGCTCTGATCACCGCGGCGTCTCTCTACGGCGCACGGCCAGACGTGGCACGGCGACCTCGC     254
33-5    TCGTTCACGTGTTACTGGTCGCTCTGATCACCGCGGCGTCTCACTACGGCGCACGGCCAGACGTGGCACGGCGACCTCGC     255
33-6    TCGTTCACGTGTTACTGGTCGCTCTGATCACCGCGGCGTCTC--TACGGCGCACGGCCAGACGTGGCACGGCGACCTCGC     253
33-7    TCGTTCACGTGTTACTGGTCGCTCTGATCACCGCGGCGTCTC--TACGGCGCACGGCCAGACGTGGCACGGCGACCTCGC     252
33-8    TCGTTCACGTGTTACTGGTCGCTCTGATCACCGCGGCGTCTCACTACGGCGCACGGCCAGACGTGGCACGGCGACCTCGC     253
33-8    TCGTTCACGTGTTACTGGTCGCTCTGATCACCGCGGCGTCTC--TACGGCGCACGGCCAGACGTGGCACGGCGACCTCGC     251
33-9    TCGTTCACGTGTTACTGGTCGCTCTGATCACCGCGGCGTCTCTCTACGGCGCACGGCCAGACGTGGCACGGCGACCTCGC     253
```

```
WT      TCGTTCACGTGTTACTGGTCGCTCTGATCACCGCGGCGTCTCC-TACGGCGCACGGCCAGACGTGGCACGGCGACCTCGC        279
34-1    TCGTTCAC-----------------------------------CTACGGCGCACGGCCAGACGTGGCACGGCGACCTCGC        218
34-2    TCGTTCAC-----------------------------------CTACKGCGSACGGCCMGACKTGGCACGGCGACCTCSC        218
34-3    TCGTTCACGTGTTACTGGTCGCTCTGATCACCGCGGCGWCTCCCTACGGCGCACGGCCAGACGTGGCACGGCGACCTCGC        254
34-4    TCGTTCACGTGTTACTGGTCGCTCTGATCACCGCGGCGTCTCCCTACGGCGCACGGCCAGACGTGGCACGGCGACCTCGC        252
34-5    TCGTTCAC-----------------------------------CTRCKGCGSACGGCCAGACKTGGCACGGCGACCTCGC        219
34-6    TCGTTCAC-----------------------------------CTACGGCGCACGGCCAGACGTGGCACGGCGACCTCGC        218
34-8    TCGTTCACGTGTTACTGGTCGCTCTGATCACCGCGGCGTCTCCCTACGGCGCACGGCCAGACGTGGCACGGCGACCTCGC        254
34-9    TCGTTCAC-----------------------------------CTRCKGCGSRYGGCCMKAMKTGGCRCGGCGACCTCSC        220
34-10   TCGTTCACGTGTTACTGGTCGCTCTGATCACCGCGGCGTCTCCCTACGGCGCACGGCCAGACGTGGCACGGCGACCTCGC        254

WT      TAATGGCGATCGTTCACGTGTTACTGGTCGCTCTGATCACCGCGGCGTCTC-CTACGGCGCACGGCCAGACGTGGCACG        269
37-1    TAATGGCGATCGTTCACGTGTTACTGGTCGCTCTGATCACCGCGGCGTCTCTCTACGGCGCACGGCCAGACGTGGCACG        242
37-2    TAATGGCGATCGTTCACGTGTTACTGGTCGCTCTGATCACCGCGGCGTCTCTCTACGGCGCACGGCCAGACGTGGCACG        245
37-3    TAATGGCGATCGTTCACGTGTTACTGGTCGCTCTGATCACCGCGGCGTCTCTCTACGGCGCACGGCCAGACGTGGCACG        243
37-4    TAATGGCGATCGTTCACGTGTTACTGGTCGCTCTGATCACCGCGGCGTCTCACTACGGCGCACGGCCAGACGTGGCACG        244
37-5    TAATGGCGATCGTTCACGTGTTACTGGTCGCTCTGATCACCGCGGCGTCTC-CTACGGCGCACGGCCAGACGTGGCACG        251
37-6    TAATGGCGATCGTTCACGTGTTACTGGTCGCTCTGATCACCGCGGCGTCTCACTACGGCGCACGGCCAGACGTGGCACG        245
37-7    TAATGGCGATCGTTCACGTGTTACTGGTCGCTCTGATCACCGCGGCGTCTCTCTACGGCGCACGGCCAGACGTGGCACG        243
37-8    TAATGGCGATCGTTCACGTGTTACTGGTCGCTCTGATCACCGCGGCGTCTCACTACGGCGCACGGCCAGACGTGGCACG        244
37-9    TAATGGCGATCGTTCACGTGTTACTGGTCGCTCTGATCACCGCGGCGTCTC-CTACGGCGCACGGCCAGACGTGGCACG        244
37-10   TAATGGCGATCGTTCACGTGTTACTGGTCGCTCTGATCACCGCGGCGTCTCACTACGGCGCACGGCCAGACGTGGCACG        244

WT      TCGTTCACGTGTTACTGGTCGCTCTGATCACCGCGGCGTCTCC-TACGGCGCACGGCCAGACGTGGCACGGCGACCTCGC        279
38-1    TCGTTCACGTGTTACTGGTCGCTCTGATCACCGCGGCGTCTCCCTACGGCGCACGGCCAGACGTGGCACGGCGACCTCGC        255
38-3    TCGTTCACGTGTTACTGGTCGCTCTGATCACCGCGGCGTCTC--TACGGCGCACGGCCAGACGTGGCACGGCGACCTCGC        252
38-4    TCGTTCACGTGTTACTGGTCGCTCTGATCACCGCGGCGTCTCTCTACGGCGCACGGCCAGACGTGGCACGGCGACCTCGC        253
38-5    TCGTTCACGTGTTACTGGTCGCTCTGATCACCGCGGCGTCTCTCTACGGCGCACGGCCAGACGTGGCACGGCGACCTCGC        254
38-6    TCGTTCACGTGTTACTGGTCGCTCTGATCACCGCGGCGTCTCT--ACGGCGCACGGCCAGACGTGGCACGGCGACCTCGC        252
38-7    TCGTTCACGTGTTACTGGTCGCTCTGATCACCGCGGCGTCTCT--ACGGCGCACGGCCAGACGTGGCACGGCGACCTCGC        252
38-8    TCGTTCACGTGTTACTGGTCGCTCTGATCACCGCGGCGTCTCT--ACGGCGCACGGCCAGACGTGGCACGGCGACCTCGC        252
38-9    TCGTTCACGTGTTACTGGTCGCTCTGATCACCGCGGCGTCTCCCTACGGCGCACGGCCAGACGTGGCACGGCGACCTCGC        254
38-10   TCGTTCACGTGTTACTGGTCGCTCTGATCACCGCGGCGTCTC--TACGGCGCACGGCCAGACGTGGCACGGCGACCTCGC        252

WT      ATCGTTCACGTGTTACTGGTCGCTCTGATCACCGCGGCGTCTC-CTACGGCGCACGGCCAGACGTGGCACGGCGACCTCG        278
41-1    ATCGTTCACGTGTTACTGGTCGCTCTGATCACCGCGGCGTCTCTCTACGGCGCACGGCCAGACGTGGCACGGCGACCTCG        253
41-2    ATCGTTCACGTGTTACTGGTCGCTCTGATCACCGCGGCGTCTCCCTACGGCGCACGGCCAGACGTGGCACGGCGACCTCG        253
41-3    ATCGTTCACGTGTTACTGGTCGCTCTGATCACCGCGGCGTCTC--TACGGCGCACGGCCAGACGTGGCACGGCGACCTCG        251
41-4    ATCGTTCACGTGTTACTGGTCGCTCTGATCACCGCGGCGTCTCCCTACGGCGCACGGCCAGACGTGGCACGGCGACCTCG        255
41-5    ATCGTTCACGTGTTACTGGTCGCTCTGATCACCGCGGCGTCTCCCTACGGCGCACGGCCAGACGTGGCACGGCGACCTCG        254
41-6    ATCGTTCACGTGTTACTGGTCGCTCTGATCACCGCGGCGTCTCCCTACGGCGCACGGCCAGACGTGGCACGGCGACCTCG        257
41-7    ATCGTTCACGTGTTACTGGTCGCTCTGATCACCGCGGCGTCTC-CTACGGCGCACGGCCAGACGTGGCACGGCGACCTCG        254
41-8    ATCGTTCACGTGTTACTGGTCGCTCTGATCACCGCGGCGTCTCACTACGGCGCACGGCCAGACGTGGCACGGCGACCTCG        253
41-9    ATCGTTCACGTGTTACTGGTCGCTCTGATCACCGCGGCGTCTCACTACGGCGCACGGCCAGACGTGGCACGGCGACCTCG        253
41-10   ATCGTTCACGTGTTACTGGTCGCTCTGATCACCGCGGCGTCTC-CTACGGCGCACGGCCAGACGTGGCACGGCGACCTCG        253
```

```
WT      TCGTTCACGTGTTACTGGTCGCTCTGATCACCGCGGCGTCTC-CTACGGCGCACGGCCAGACGTGGCACGGCGACCTCGC        279
42-1    TCGTTCACGTGTTACTGGTCGCTCTGATCACCGCGGCGTCTCTCTACGGCGCACGGCCAGACGTGGCACGGCGACCTCGC        253
42-2    TCGTTCACGTGTTACTGGTCGCTCTGATCACCGCGGCGTCTCTCTACGGCGCACGGCCAGACGTGGCACGGCGACCTCGC        254
42-3    TCGTTCACGTGTTACTGGTCGCTCTGATCACCGCGGCGTCTCACTACGGCGCACGGCCAGACGTGGCACGGCGACCTCGC        255
42-5    TCGTTCACGTGTTACTGGTCGCTCTGATCACCGCGGCGTCTCTCTACGGCGCACGGCCAGACGTGGCACGGCGACCTCGC        253
42-6    TCGTTCACGTGTTACTGGTCGCTCTGATCACCGCGGCGTCTCACTACGGCGCACGGCCAGACGTGGCACGGCGACCTCGC        254
42-7    TCGTTCACGTGTTACTGGTCGCTCTGATCACCGCGGCGTCTCTCTACGGCGCACGGCCAGACGTGGCACGGCGACCTCGC        255
42-8    TCGTTCACGTGTTACTGGTCGCTCTGATCACCGCGGCGTCTC--TACGGCGCACGGCCAGACGTGGCACGGCGACCTCGC        251
42-9    TCGTTCACGTGTTACTGGTCGCTCTGATCACCGCGGCGTCTC--TACGGCGCACGGCCAGACGTGGCACGGCGACCTCGC        251
42-10   TCGTTCACGTGTTACTGGTCGCTCTGATCACCGCGGCGTCTC--TACGGCGCACGGCCAGACGTGGCACGGCGACCTCGC        252

WT      GATCGTTCACGTGTTACTGGTCGCTCTGATCACCGCGGCGTCTCC-TACGGCGCACGGCCAGACGTGGCACGGCGACCTC        277
44-9    GATCGTTCACGTGTTACTGGTCGCTCTGATCACCGCGGCGTCTCCCTACGGCGCACGGCCAGACGTGGCACGGCGACCTC        252
44-2    GATCGTTCACGTGTTACTGGTCGCTCTGATCACCGCGGCGTCTCC-TACGGCGCACGGCCAGACGTGGCACGGCGACCTC        252
44-10   GATCGTTCACGTGTTACTGGTCGCTCTGATCACCGCGGCGTCTCC-TACGGCGCACGGCCAGACGTGGCACGGCGACCTC        251
44-4    GATCGTTCACGTGTTACTGGTCGCTCTGATCACCGCGGCGTCTCC-TACGGCGCACGGCCAGACGTGGCACGGCGACCTC        252
44-5    GATCGTTCACGTGTTACTGGTCGCTCTGATCACCGCGGCGTCTC--TACGGCGCACGGCCAGACGTGGCACGGCGACCTC        249
44-6    GATCGTTCACGTGTTACTGGTCGCTCTGATCACCGCGGCGTCTC--TACGGCGCACGGCCAGACGTGGCACGGCGACCTC        249
44-1    GATCGTTCACGTGTTACTGGTCGCTCTGATCACCGCGGCGTCTC--TACGGCGCACGGCCAGACGTGGCACGGCGACCTC        249
44-3    GATCGTTCACGTGTTACTGGTCGCTCTGATCACCGCGGCGTCTC--TACGGCGCACGGCCAGACGTGGCACGGCGACCTC        249
44-7    GATCGTTCACGTGTTACTGGTCGCTCTGATCACCGCGGCGTCTC--TACGGCGCACGGCCAGACGTGGCACGGCGACCTC        250
44-8    GATCGTTCACGTGTTACTGGTCGCTCTGATCACCGCGGCGTCTC--TACGGCGCACGGCCAGACGTGGCACGGCGACCTC        250

WT      TCGTTCACGTGTTACTGGTCGCTCTGATCACCGCGGCGTCTC-CTACGGCGCACGGCCAGACGTGGCACGGCGACCTCGC        279
45-10   TCGTTCACGTGTTACTGGTCGCTCTGATCACCGCGGCGTCTCCCTACGGCGCACGGCCAGACGTGGCACGGCGACCTCGC        256
45-1    TCGTTCACGTGTTACTGGTCGCTCTGATCACCGCGGCGTCTCACTACGGCGCACGGCCAGACGTGGCACGGCGACCTCGC        253
45-2    TCGTTCACGTGTTACTGGTCGCTCTGATCACCGCGGCGTCTCACTACGGCGCACGGCCAGACGTGGCACGGCGACCTCGC        253
45-3    TCGTTCACGTGTTACTGGTCGCTCTGATCACCGCGGCGTCTCCCTACGGCGCACGGCCAGACGTGGCACGGCGACCTCGC        254
45-4    TCGTTCACGTGTTACTGGTCGCTCTGATCACCGCGGCGTCTCCCTACGGCGCACGGCCAGACGTGGCACGGCGACCTCGC        254
45-5    TCGTTCACGTGTTACTGGTCGCTCTGATCACCGCGGCGTCTCCCTACGGCGCACGGCCAGACGTGGCACGGCGACCTCGC        254
45-6    TCGTTCACGTGTTACTGGTCGCTCTGATCACCGCGGCGTCTCCCTACGGCGCACGGCCAGACGTGGCACGGCGACCTCGC        254
45-7    TCGTTCACGTGTTACTGGTCGCTCTGATCACCGCGGCGTCTCCCTACGGCGCACGGCCAGACGTGGCACGGCGACCTCGC        254
45-8    TCGTTCACGTGTTACTGGTCGCTCTGATCACCGCGGCGTCTCCCTACGGCGCACGGCCAGACGTGGCACGGCGACCTCGC        254
45-9    TCGTTCACGTGTTACTGGTCGCTCTGATCACCGCGGCGTCTC--TACGGCGCACGGCCAGACGTGGCACGGCGACCTCGC        252

WT      CGATCGTTCACGTGTTACTGGTCGCTCTGATCACCGCGGCGTCTC-CTACGGCGCACGGCCAGACGTGGCACGGCGACCT        276
47-9    CGATCGTTCACGTGTTACTGGTCGCTCTGATCACCGCGGCGTCTCCCTACGGCGCACGGCCAGACGTGGCACGGCGACCT        250
47-1    CGATCGTTCACGTGTTACTGGTCGCTCTGATCACCGCGGCGTCTCACTACGGCGCACGGCCAGACGTGGCACGGCGACCT        250
47-2    CGATCGTTCACGTGTTACTGGTCGCTCTGATCACCGCGGCGTCT--CTACGGCGCACGGCCAGACGTGGCACGGCGACCT        249
47-3    CGATCGTTCACGTGTTACTGGTCGCTCTGATCACCGCGGCGTCTCCCTACGGGCAMGGGCCRAACTGGGCACGGCAACTT        252
47-4    CGATCGTTCACGTGTTACTGGTCGCTCTGATCACCGCGGCGTCTCCCTACGGCGCACGGCCAGACGTGGCACGGCGACCT        251
47-5    CGATCGTTCACGTGTTACTGGTCGCTCTGATCACCGCGGCGTCTCCCTACGGCGCACGGCCAGACGTGGCACGGCGACCT        251
47-6    CGATCGTTCACGTGTTACTGGTCGCTCTGATCACCGCGGCGTCT--CTACGGCGCACGGCCAGACGTGGCACGGCGACCT        248
47-7    CGATCGTTCACGTGTTACTGGTCGCTCTGATCACCGCGGCGTCT--CTACGGCGCACGGCCAGACGTGGCACGGCGACCT        248
47-8    CGATCGTTCACGTGTTACTGGTCGCTCTGATCACCGCGGCGTCTCACTACGGCGCACGGCCAGACGTGGCACGGCGACCT        251
```

```
WT      ATCGTTCACGTGTTACTGGTCGCTCTGATCACCGCGGCGTCTCC-TACGGCGCACGGCCAGACGTGGCACGGCGACCTCG        278
54-10   ATCGTTCACGTGTTACTGGTCGCTCTGATCACCGCGGCGTC------CGGCGCACGGCCAGACGTGGCACGGCGACCTCG        248
54-1    ATCGTTCACGTGTTACTGGTCGCTCTGATCACCGCGGCGTC------CGGCGCACGGCCAGACGTGGCACGGCGACCTCG        246
54-2    ATCGTTCACGTGTTACTGGTCGCTCTGATCACCGCGGCGTCTC--TACGGCGCACGGCCAGACGTGGCACGGCGACCTCG        254
54-3    ATCGTTCACGTGTTACTGGTCGCTCTGATCACCGCGGCGTCTC--TACGGCGCACGGCCAGACGTGGCACGGCGACCTCG        251
54-4    ATCGTTCACGTGTTACTGGTCGCTCTGATCACCGCGGCGTCTC--TMSCRCGSACRGACRTASSTGGSMSACCTACCTCC        249
54-5    ATCGTTCACGTGTTACTGGTCGCTCTGATCACCGCGGCGTCTC--TACGGCGCACGGCCAGACGTGGCACGGCGACCTCG        254
54-6    ATCGTTCACGTGTTACTGGTCGCTCTGATCACCGCGGCGTC------CGGCGCACGGCCAGACGTGGCACGGCGACCTCG        249
54-7    ATCGTTCACGTGTTACTGGTCGCTCTGATCACCGCGGCGTCTC--TACGGCGCACGGCCAGACGTGGCACGGCGACCTCG        254
54-8    ATCGTTCACGTGTTACTGGTCGCTCTGATCACCGCGGCGTCTC--TACGGCGCACGGCCAGACGTGGCACGGCGACCTCG        252
54-9    ATCGTTCACGTGTTACTGGTCGCTCTGATCACCGCGGCGTCTC--TACGGCGCACGGCCAGACGTGGCACGGCGACCTCG        251
```

```
WT      TCGTTCACGTGTTACTGGTCGCTCTGATCACCGCGGCGTCTC-CTACGGCGCACGGCCAGACGTGGCACGGCGACCTCGC        279
57-10   TCGTTCACGTGTTACTGGTCGCTCTGATCACCGCGGCGTCTCACTACGGCGCACGGCCAGACGTGGCACGGCGACCTCGC        255
57-1    TCGTTCACGTGTTACTGGTCGCTCTGATCACCGCGGCGT----CTACGGCGCACGGCCAGACGTGGCACGGCGACCTCGC        252
57-2    TCGTTCACGTGTTACTGGTCGCTCTGATCACCGCGGCGTCTCACTACGGCGCACGGCCAGACGTGGCACGGCGACCTCGC        253
57-7    TCGTTCACGTGTTACTGGTCGCTCTGATCACCGCGGCGT----CTACSGMGCACGGMCAGACGTGGCACGGCGACCTCSC        252
57-3    TCGTTCACGTGTTACTGGTCGCTCTGATCACCGCGGCGTCTCMCKACSGCGSACGGMCAKACSTGGSACGGCKACCTCSC        255
57-4    TCGTTCACGTGTTACTGGTCGCTCTGATCACCGCGGCGTCTCACTACGGCGCACGGCCAGACGTGGCACGGCGACCTCGC        254
57-5    TCGTTCACGTGTTACTGGTCGCTCTGATCACCGCGGCGTCTCACTACGGCGCACGGCCAGACGTGGCACGGCGACCTCGC        255
57-6    TCGTTCACGTGTTACTGGTCGCTCTGATCACCGCGGCGTCTCACTACGGCGCACGGCCAGACGTGGCACGGCGACCTCGC        254
57-8    TCGTTCACGTGTTACTGGTCGCTCTGATCACCGCGGCGTCTCACTACGGCGCACGGCCAGACGTGGCACGGCGACCTCGC        253
57-9    TCGTTCACGTGTTACTGGTCGCTCTGATCACCGCGGCGTCTCACTACGGCGCACGGCCAGACGTGGCACGGCGACCTCGC        255
```

```
WT      TCGTTCACGTGTTACTGGTCGCTCTGATCACCGCGGCGTCTC-CTACGGCGCACGGCCAGACGTGGCACGGCGACCTCGC        279
63-10   TCGTTCACGTGTTACTGGTCGCTCTGATCACCGCGGCGTCTCACTACGGCGCACGGCCAGACGTGGCACGGCGACCTCGC        254
63-1    TCGTTCACGTGTTACTGGTCGCTCTGATCACCGCGGCGTCTCACTACGGCGCACGGCCAGACGTGGCACGGCGACCTCGC        254
63-2    TCGTTCACGTGTTACTGGTCGCTCTGATCACCGCGGCGTCTCACTACGGCGCACGGCCAGACGTGGCACGGCGACCTCGC        253
63-3    TCGTTCACGTGTTACTGGTCGCTCTGATCACCGCGGCGTCTCACTACGGCGCACGGCCAGACGTGGCACGGCGACCTCGC        253
63-4    TCGTTCACGTGTTACTGGTCGCTCTGATCACCGCGGCGTCTCACTACCGCGCACGGACAGACGTGGCACGGCKACCTCSC        253
63-5    TCGTTCACGTGTTACTGGTCGCTCTGATCACCGCGGCGTCTCACTACGGCGCACGGCCAGACGTGGCACGGCGACCTCGC        253
63-6    TCGTTCACGTGTTACTGGTCGCTCTGATCACCGCGGCGTCTCACTACGGCGCACGGCCAGACGTGGCACGGCGACCTCGC        252
63-7    TCGTTCACGTGTTACTGGTCGCTCTGATCACCGCGGCGTCTCACTACGGCGCACGGCCAGACGTGGCACGGCGACCTCGC        254
63-8    TCGTTCACGTGTTACTGGTCGCTCTGATCACCGCGGCGTCTCACTACGGCGCACGGCCAGACGTGGCACGGCGACCTCGC        254
63-9    TCGTTCACGTGTTACTGGTCGCTCTGATCACCGCGGCGTCTCACTACGGCGCACGGCCAGACGTGGCACGGCGACCTCGC        254
```

```
WT      TCGTTCACGTGTTACTGGTCGCTCTGATCACCGCGGCGTCTC-CTACGGCGCACGGCCAGACGTGGCACGGCGACCTCGC        279
67-10   TCGTTCACGTGTTACTGGTCGCTCTGATCACCGCGGCGTCT--CTACGGCGCACGGCCAGACGTGGCACGGCGACCTCGC        252
67-1    TCGTTCACGTGTTACTGGTCGCTCTGATCACCGCGGCGTCTCACTACGGCGCACGGCCAGACGTGGCACGGCGACCTCGC        253
67-2    TCGTTCACGTGTTACTGGTCGCTCTGATCACCGCGGCGTCTC--TACGGCGCACGGCCAGACGTGGCACGGCGACCTCGC        251
67-3    TCGTTCACGTGTTACTGGTCGCTCTGATCACCGCGGCGTCTCACTACGGCGCACGGCCAGACGTGGCACGGCGACCTCGC        254
67-4    TCGTTCACGTGTTACTGGTCGCTCTGATCACCGCGGCGTCT--CTACGGCGCACGGCCAGACGTGGCACGGCGACCTCGC        252
67-5    TCGTTCACGTGTTACTGGTCGCTCTGATCACCGCGGCGTCT--CTACGGCGCACGGCCAGACGTGGCACGGCGACCTCGC        252
67-6    TCGTTCACGTGTTACTGGTCGCTCTGATCACCGCGGCGTCTCACTACGGCGCACGGCCAGACGTGGCACGGCGACCTCGC        253
67-7    TCGTTCACGTGTTACTGGTCGCTCTGATCACCGCGGCGTC------------------------------------CTCGC        219
67-8    TCGTTCACGTGTTACTGGTCGCTCTGATCACCGCGGCGTCTCACTACGGCGCACGGCCAGACGTGGCACGGCGACCTCGC        253
67-9    TCGTTCACGTGTTACTGGTCGCTCTGATCACCGCGGCGTCTCCCTACGGCGCACGGCCAGACGTGGCACGGCGACCTCGC        255
```

```
WT      ATCGTTCACGTGTTACTGGTCGCTCTGATCACCGCGGCGTCTC-CTACGGCGCACGGCCAGACGTGGCACGGCGACCTCG         278
70-10   ATCGTTCACGTGTTACTGGTCGCTCTGATCACCGCGGCGTCTC--TACGGCGCACGGCCAGACGTGGCACGGCGACCTCG         252
70-1    ATCGTTCACGTGTTACTGGTCGCTCTGATCACCGCGGCGTCTCCCTACGGCGCACGGCCAGACGTGGCACGGCGACCTCG         252
70-2    ATCGTTCACGTGTTACTGGTCGCTCTGATCACCGCGGCGTCTCCCTACGGCGCACGGCCAGACGTGGCACGGCGACCTCG         253
70-3    ATCGTTCACGTGTTACTGGTCGCTCTGATCACCGCGGCGTCTC--TACGGCGCACGGCCAGACGTGGCACGGCGACCTCG         250
70-4    ATCGTTCACGTGTTACTGGTCGCTCTGATCACCGCGGCGTCTCCCTACGGCGCACGGCCAGACGTGGCACGGCGACCTCG         253
70-5    ATCGTTCACGTGTTACTGGTCGCTCTGATCACCGCGGCGTCTCCCTACGGCGCACGGCCAGACGTGGCACGGCGACCTCG         252
70-6    ATCGTTCACGTGTTACTGGTCGCTCTGATCACCGCGGCGTCTC--TACGGCGCACGGCCAGACGTGGCACGGCGACCTCG         250
70-7    ATCGTTCACGTGTTACTGGTCGCTCTGATCACCGCGGCGTCTC--TACGGCGCACGGCCAGACGTGGCACGGCGACCTCG         253
70-8    ATCGTTCACGTGTTACTGGTCGCTCTGATCACCGCGGCGTCTCCCTACGGCGCACGGCCAGACGTGGCACGGCGACCTCG         252
70-9    ATCGTTCACGTGTTACTGGTCGCTCTGATCACCGCGGCGTCT--CTACGGCGCACGGCCAGACGTGGCACGGCGACCTCG         250

WT      TCGTTCACGTGTTACTGGTCGCTCTGATCACCGCGGCGTCTC-CTACGGCGCACGGCCAGACGTGGCACGGCGACCTCGC         279
78-10   TCGTTCACGTGTTACTGGTCGCTCTGATCACCGCGGCGTCTC--TACGGCGCACGGCCAGACGTGGCACGGCGACCTCGC         248
78-1    TCGTTCACGTGTTACTGGTCGCTCTGATCACCGCGGCGTCTCACTACGGCGCACGGCCAGACGTGGCACGGCGACCTCGC         254
78-2    TCGTTCACGTGTTACTGGTCGCTCTGATCACCGCGGCGTCTCACTACGGCGCACGGCCAGACGTGGCACGGCGACCTCGC         253
78-3    TCGTTCACGTGTTACTGGTCGCTCTGATCACCGCGGCGTCTCACTACGGCGCACGGCCAGACGTGGCACGGCGACCTCGC         254
78-4    TCGTTCACGTGTTACTGGTCGCTCTGATCACCGCGGCGTCTC--TACGGCGCACGGCCAGACGTGGCACGGCGACCTCGC         252
78-5    TCGTTCACGTGTTACTGGTCGCTCTGATCACCGCGGCGTCT--------------------------GGCKACCKCGC         227
78-6    TCGTTCACGTGTTACTGGTCGCTCTGATCACCGCGGCGTCTC--TACGGCGCACGGCCAGACGTGGCACGGCGACCTCGC         251
78-7    TCGTTCACGTGTTACTGGTCGCTCTGATCACCGCGGCGTCTC--TACGGCGCACGGCCAGACGTGGCACGGCGACCTCGC         252
78-8    TCGTTCACGTGTTACTGGTCGCTCTGATCACCGCGGCGTCTCACTACGGCGCACGGCCAGACGTGGCACGGCGACCTCGC         249
78-9    TCGTTCACGTGTTACTGGTCGCTCTGATCACCGCGGCGTCTCACTACGGCGCACGGCCAGACGTGGCACGGCGACCTCGC         251

WT      TCGTTCACGTGTTACTGGTCGCTCTGATCACCGCGGCGTCTCC-TACGGCGCACGGCCAGACGTGGCACGGCGACCTCGC         279
80-10   TCGTTCACGTGTTACTGGTCGCTCTGATCACCGCGGCGTC-CT--ACGGCGCACGGCCAGACGTGGCACGGCGACCTCGC         248
80-1    TCGTTCACGTGTTACTGGTCGCTCTGATCACCGCGGCGTCTCTCTACGGCGCACGGCCAGACGTGGCACGGCGACCTCGC         249
80-2    TCGTTCACGTGTTACTGGTCGCTCTGATCACCGCGGCGTCTCT--ACGGCGCACGGCCAGACGTGGCACGGCGACCTCGC         246
80-3    TCGTTCACGTGTTACTGGTCGCTCTGATCACCGCGGCGTCTCT--ACGGCGCACGGCCAGACGTGGCACGGCGACCTCGC         248
80-4    TCGTTCACGTGTTACTGGTCGCTCTGATCACCGCGGCGTCTCT--ACGGCGCACGGCCAGACGTGGCACGGCGACCTCGC         248
80-5    TCGTTCACGTGTTACTGGTCGCTCTGATCACCGCGGCGTCTCT--ACGGCGCACGGCCAGACGTGGCACGGCGACCTCGC         248
80-6    TCGTTCACGTGTTACTGGTCGCTCTGATCACCGCGGCGTCTCT--ACGGCGCACGGCCAGACGTGGCACGGCGACCTCGC         248
80-7    TCGTTCACGTGTTACTGGTCGCTCTGATCACCGCGGCGTCTCT--ACGGCGCACGGCCAGACGTGGCACGGCGACCTCGC         248
80-8    TCGTTCACGTGTTACTGGTCGCTCTGATCACCGCGGCGTCTCT--ACGGCGCACGGCCAGACGTGGCACGGCGACCTCGC         248
80-9    TCGTTCACGTGTTACTGGTCGCTCTGATCACCGCGGCGTCTCT--ACGGCGCACGGCCAGACGTGGCACGGCGACCTCGC         248
```

Fig. S3 Sequence alignment of the *HvCKX1* gene fragments cloned from selected T_0 plants. Target sequence is marked in yellow and PAM motif in light blue; deletions are indicated by dashes.
