# Supplementary material for: A simple and efficient CRISPR/Cas9 platform for induction of single and multiple, heritable mutations in barley (Hordeum vulgare L.)
Source: Plant Methods. 2018 Dec 18;14:111. doi: 10.1186/s13007-018-0382-8 (PMC6297969; doi:10.1186/s13007-018-0382-8)
Supplement: Supplementary file 3 — Additional file 3: Figure S4. Sequence alignment of the HvCKX1 gene fragments cloned from selected T1 plants. Target sequence is marked in yellow and PAM motif in light blue; deletions are indicated by dashes. [file 13007_2018_382_MOESM3_ESM.pdf]

Fig. S4

```
WT      TCGTTCACGTGTTACTGGTCGCTCTGATCACCGCGGCGTCTCCTACGGCGCACGGCCAGACGTGGCACGGCGACCTCGCG        280
22-1    TCGTTCACGTGTTACTGGTCGCTCTGATCACC-------------------------AGACGTGGCACGGCGACCTCGCG        229
22-3    TCGTTCACGTGTTACTGGTCGCTCTGATCACC-------------------------AGACGTGGCACGGCGACCTCGCG        227
22-5    TCGTTCACGTGTTACTGGTCGCTCTGATCACC-------------------------AGACGTGGCACGGCGACCTCGCG        228
22-6    TCGTTCACGTGTTACTGGTCGCTCTGATCACC-------------------------AGACGTGGCACGGCGACCTCGCG        227
22-7    TCGTTCACGTGTTACTGGTCGCTCTGATCACC-------------------------AGACGTGGCACGGCGACCTCGCG        230

WT      TCGTTCACGTGTTACTGGTCGCTCTGATCACCGCGGCGTCTC-CTACGGCGCA-CGGCCAGACGTGGCACGGCGACCTCG        278
27-1    TCGTTCACGTGTTACTGGTCGCTCTGATCACCGCGGTACGCCGCCGTGCCACGGCGACCAGACGTGGCACGGCGACCTCG        253
27-5    TCGTTCACGTGTTACTGGTCGCTCTGATCACC---------------------------AGACGTGGCACGGCGACCTCG        225

WT      TCGTTCACGTGTTACTGGTCGCTCTGATCACCGCGGCGTCTCCTACGGCGCACGGCCAGACGTGGCACGGCGACCTCGCG        280
29-8    TCGTTCACGTGTTACTGGTCGCTCTGATCACC-----------TACGGCGCACGGCCAGACGTGGCACGGCGACCTCGCG        242
29-3    TCGTTCACGTGTTACTGGTCGCTCTGATCACC-----------TACGGCGCACGGCCAGACGTGGCACGGCGACCTCGCG        243

WT      TCGTTCACGTGTTACTGGTCGCTCTGATCACCGCGGCGTCTCCTACGGCGCACGGCCAGACGTGGCACGGCGACCTCGCG        280
34-1    TCGTTCAC----------------------------------CTACGGCGCACGGCCAGACGTGGCACGGCGACCTCGCG        220
34-2    TCGTTCAC----------------------------------CTACGGCGCACGGCCAGACGTGGCACGGCGACCTCGCG        219
34-3    TCGTTCAC----------------------------------CTACGGCGCACGGCCAGACGTGGCACGGCGACCTCGCG        219
34-5    TCGTTCAC----------------------------------CTACGGCGCACGGCCAGACGTGGCACGGCGACCTCGCG        218
34-6    TCGTTCAC----------------------------------CTACGGCGCACGGCCAGACGTGGCACGGCGACCTCGCG        220
34-7    TCGTTCAC----------------------------------CTACGGCGCACGGCCAGACGTGGCACGGCGACCTCGCG        219
34-8    TCGTTCAC----------------------------------CTACGGCGCACGGCCAGACGTGGCACGGCGACCTCGCG        220
34-9    TCGTTCAC----------------------------------CTACGGCGCACGGCCAGACGTGGCACGGCGACCTCGCG        219
34-10   TCGTTCAC----------------------------------CTACGGCGCACGGCCAGACGTGGCACGGCGACCTCGCG        219

WT      TCGTTCACGTGTTACTGGTCGCTCTGATCACCGCGGCGTCTCCTACGGCGCACGGCCAGACGTGGCACGGCGACCTCGCG        280
54-4    TCGTTCACGTGTTACTGGTCGCTCTGATCACCGCGGCGTCT-----GGCGCACGGCCAGACGTGGCACGGCGACCTCGCG        248

WT      TCGTTCACGTGTTACTGGTCGCTCTGATCACCGCGGCGTCTCCTACGGCGCACGGCCAGACGTGGCACGGCGACCTCGCG        280
57-6    TCGTTCACGTGTTACTGGTCGCTCTGATCACCGCGGCGTCT---ACGGCGCACGGCCAGACGTGGCACGGCGACCTCGCG        251
57-7    TCGTTCACGTGTTACTGGTCGCTCTGATCACCGCGGCGTCT---ACGGCGCACGGCCAGACGTGGCACGGCGACCTCGCG        250
57-9    TCGTTCACGTGTTACTGGTCGCTCTGATCACCGCGGCGTCT---ACGGCGCACGGCCAGACGTGGCACGGCGACCTCGCG        251

WT      TCGTTCACGTGTTACTGGTCGCTCTGATCACCGCGGCGTCTCCTACGGCGCACGGCCAGACGTGGCACGGCGACCTCGCG        280
63-1    TCGTTCACGTGTTACTGGTCGCTCTGATCACCGCGGCGTCT---ACGGCGCACGGCCAGACGTGGCACGGCGACCTCGCG        250
63-6    TCGTTCACGTGTTACTGGTCGCTCTGATCACCGCGGCGTCT---ACGGCGCACGGCCAGACGTGGCACGGCGACCTCGCG        251
```

```
WT      TCGTTCACGTGTTACTGGTCGCTCTGATCACCGCGGCGTCTCCTACGGCGCACGGCCAGACGTGGCACGGCGACCTCGCG        280
78-1    TCGTTCACGTGTTACTGGTCGCTCTGATCACCGCGGCGTCT---------------------------GGCGACCTCGCG        228
78-5    TCGTTCACGTGTTACTGGTCGCTCTGATCACCGCGGCGTCT---------------------------GGCGACCTCGCG        228
```

Fig. S4 Sequence alignment of the *HvCKX1* gene fragments cloned from selected T_1 plants. Target sequence is marked in yellow and PAM motif in light blue; deletions are indicated by dashes.
