# Supplementary material for: A simple and efficient CRISPR/Cas9 platform for induction of single and multiple, heritable mutations in barley (Hordeum vulgare L.)
Source: Plant Methods. 2018 Dec 18;14:111. doi: 10.1186/s13007-018-0382-8 (PMC6297969; doi:10.1186/s13007-018-0382-8)
Supplement: Supplementary file 4 — Additional file 4: Figure S5. Sequence alignment of the Nud gene fragments cloned from selected T0 plants. Target sequence is marked in yellow and PAM motif in light blue; deletions are indicated by dashes. [file 13007_2018_382_MOESM4_ESM.pdf]

**Fig. S5**

```
WT     TACAGGAAGAGGAGGGTGTGGTTGGGCACCTTTGAGACGGCGGAGGAGGCTGCGCGGGCGTACGATGAGGCTGCCATCC    348
14-1   TWCMGGARGAGG----------------------------------AGGCTGCGCGGGCGTACGATGAGGCTGCCATCC    337
14-2   TACAGGAAGAGG----------------------------------AGGCTGCGCGGGCGTACGATGAGGCTGCCATCC    338
14-3   TACAGGAAGAGGAGGGTGTGGTTGGGCACCTTTG---CGGCGGAGGAGGCTGCGCGGGCGTACGATGAGGCTGCCATCC    388
14-4   TACACGARAAGAAGAGGGTGCKTGGGCRCCTTAG---CAGGSTACCATCCTGATCAGSGGKACCAWGASGCTGACCTCC    338

WT     TACAGGAAGAGGAGGGTGTGGTTGGGCACCTTTGAGAC-GGCGGAGGAGGCTGCGCGGGCGTACGATGAGGCTGCCATCC   348
20-1   TACAGGAAGAGGAGGGTGTGGTTGGGCACCTTTGAGAC-GGCGGAGGAGGCTGCGCGGGCGTACGATGAGGCTGCCATCC   202
20-2   TACAGGAAGAGGAGGGTGTGGTTGGGCACCTTTGAGAC-GGCGGAGGAGGCTGCGCGGGCGTACGATGAGGCTGCCATCC   202
20-3   TACAGGAAGAGGAGGGTGTGGTTGGGCACCTTTGAGAC-GGCGGAGGAGGCTGCGCGGGCGTACGATGAGGCTGCCATCC   202
20-4   TACAGGAAGAGGAGGGTGTGGTTGGGCACCTTTGAGACCGGCGGAGGAGGCTGCGCGGGCGTACGATGAGGCTGCCATCC   203
20-5   TACAGGAAGAGGAGGGTGTGGTTGGGCACCTTTGAGAC-GGCGGAGGAGGCTGCGCGGGCGTACGATGAGGCTGCCATCC   202

WT     TACAGGAAGAGGAGGGTGTGGTTGGGCACCTTTGAGA-CGGCGGAGGAGGCTGCGCGGGCGTACGATGAGGCTGCCATCC   348
43-1   TACAGGAAGAGGAGGGTGTGGTTGGGCACCTTTGAGATCGGCGGAGGAGGCTGCGCGGGCGTACGATGAGGCTGCCATCC   167
43-2   TACAGGAAGAGGAGGGTGTGGTTGGGCACCTTTGAGATCGGCGGAGGAGGCTGCGCGGGCGTACGATGAGGCTGCCATCC   169
43-3   TACAGGAAGAGGAGGGTGTGGTTGGGCACCTTTGAGATCGGCGGAGGAGGCTGCGCGGGCGTACGATGAGGCTGCCATCC   169
43-4   TACAGGAAGAGGAGGGTGTGGTTGGGCACCTTTGAGATCGGCGGAGGAGGCTGCGCGGGCGTACGATGAGGCTGCCATCC   170
43-5   TACAGGAAGAGGAGGGTGTGGTTGGGCACCTTTGAGATCGGCGGAGGAGGCTGCGCGGGCGTACGATGAGGCTGCCATCC   169

WT     TACAGGAAGAGGAGGGTGTGGTTGGGCACCTTTGAGA-CGGCGGAGGAGGCTGCGCGGGCGTACGATGAGGCTGCCATCC   348
83-1   TACAGGAAGAGGAGGGTGTGGTTGGGCACCTTTGAGA-CGGCGGAGGAGGCTGCGCGGGCGTACGATGAGGCTGCCATCC   159
83-3   TACAGGAAGAGGAGGGTGTGGTTGGGCACCTTTG-------CGGAGGAGGCTGCGCGGGCGTACGATGAGGCTGCCATCC   153
83-4   TACAGGAAGAGGAGGGTGTGGTTGGGCACCTTTGAGATCGGCGGAGGAGGCTGCGCGGGCGTACGATGAGGCTGCCATCC   160

WT     TACAGGAAGAGGAGGGTGTGGTTGGGCACCTTTGAGA-CGGCGGAGGAGGCTGCGCGGGCGTACGATGAGGCTGCCATCC   348
85-1   TACAGGAAGAGGAGGGTGTGGTTGGGCACCTTTGAGACCGGCGGAGGAGGCTGCGCGGGCGTACGATGAGGCTGCCATCC   160
85-2   TACAGGAAGAGGAGGGTGTGGTTGGGCACCTTTGAGAACGGCGGAGGAGGCTGCGCGGGCGTACGATGAGGCTGCCATCC   160
85-3   TACAGGAAGAGGAGGGTGTGGTTGGGCACCTTTGAGACCGGCGGAGGAGGCTGCGCGGGCGTACGATGAGGCTGCCATCC   160
85-4   TACAGGAAGAGGAGGGTGTGGTTGGGCACCTTTGAGACCGGCGGAGGAGGCTGCGCGGGCGTACGATGAGGCTGCCATCC   160
85-5   TACAGGAAGAGGAGGGTGTGGTTGGGCACCTTTGAGACCGGCGGAGGAGGCTGCGCGGGCGTACGATGAGGCTGCCATCC   159
```

```
WT     TACAGGAAGAGGAGGGTGTGGTTGGGCACCTTTGAGACGGCGGAGGAGGCTGCGCGGGCGTACGATGAGGCTGCCATCC    348
86-1   TACAGGAAGAGGAGGGTGTGGTTGGGCACCTTTGAGACGGCGGAGGAGGCTGCGCGGGCGTACGATGAGGCTGCCATCC    160
86-2   TACAGGAAGAGGAGGGTGTGGTTGGGCACCTTTGAG----------AGGCTGCGCGGGCGTACGATGAGGCTGCCATCC    150
86-3   TACAGGAAGAGGAGGGTGTGGTTGGGCACCTTTGAGACGGCGGAGGAGGCTGCGCGGGCGTACGATGAGGCTGCCATCC    160

WT     TACAGGAAGAGGAGGGTGTGGTTGGGCACCTTTGAGA-CGGCGGAGGAGGCTGCGCGGGCGTACGATGAGGCTGCCATCC   348
89-1   TACAGGAAGAGGAGGGTGTGGTTGGGCACCTTTG----CGGCGGAGGAGGCTGCGCGGGCGTACGATGAGGCTGCCATCC   157
89-2   TACAGGAAGAGGAGGGTGTGGTTGGGCACCTTTGAGATCGGCGGAGGAGGCTGCGCGGGCGTACGATGAGGCTGCCATCC   161
89-3   TACAGGAAGAGGAGGGTGTGGTTGGGCACCTTTGAGA-CGGCGGAGGAGGCTGCGCGGGCGTACGATGAGGCTGCCATCC   160
89-4   TACAGGAAGAGGAGGGTGTGGTTGGGCACCTTTGAGA-CGGCGGAGGAGGCTGCGCGGGCGTACGATGAGGCTGCCATCC   160
89-5   TACAGGAAGAGGAGGGTGTGGTTGGGCACCTTTGAGATCGGCGGAGGAGGCTGCGCGGGCGTACGATGAGGCTGCCATCC   162
```

**Fig. S5** Sequence alignment of the *Nud* gene fragments cloned from selected $T_0$ plants. Target sequence is marked in yellow and PAM motif in light blue; deletions are indicated by dashes.
